# Large Biaxial Compressive Strain Tuning of Neutral and Charged Excitons in Single-Layer Transition Metal Dichalcogenides

**DOI:** 10.1021/acsami.3c13281

**Published:** 2023-11-30

**Authors:** Eudomar Henríquez-Guerra, Hao Li, Pablo Pasqués-Gramage, Daniel Gosálbez-Martínez, Roberto D’Agosta, Andres Castellanos-Gomez, M. Reyes Calvo

**Affiliations:** † Departamento de Física Aplicada, Universidad de Alicante, 03690 Alicante, Spain; ‡ Instituto Universitario de Materiales IUMA, Universidad de Alicante, 03690 Alicante, Spain; § Materials Science Factory, Instituto de Ciencia de Materiales de Madrid, Consejo Superior de Investigaciones Científicas, 28049 Madrid, Spain; ∥ Nano-bio Spectroscopy Group and European Theoretical Spectroscopy Facility (ETSF), Departamento de Polímeros y Materiales Avanzados: Física, Química y Tecnología, Universidad del Pais Vasco (UPV/EHU), E-20018 San Sebastián, Spain; ⊥ IKERBASQUE, Basque Foundation for Science, E-48013 Bilbao, Spain

**Keywords:** 2D materials, transition metal dichalcogenides, biaxial compressive strain, micro-reflectance spectroscopy, differential reflectance, excitons, trions, binding energy

## Abstract

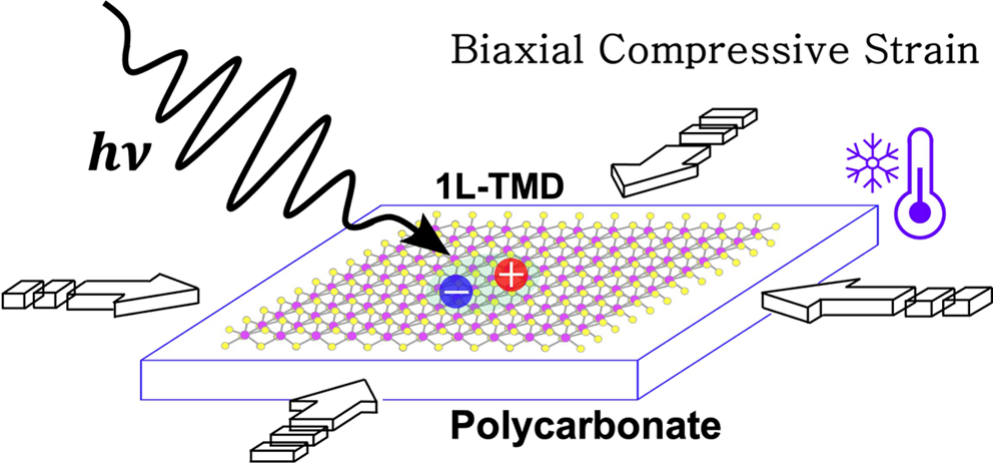

The absorption and emission of light in single-layer transition metal dichalcogenides are governed by the formation of excitonic quasiparticles. Strain provides a powerful technique to tune the optoelectronic properties of two-dimensional materials and thus to adjust their exciton energies. The effects of large compressive strain in the optical spectrum of two-dimensional (2D) semiconductors remain rather unexplored compared to those of tensile strain, mainly due to experimental constraints. Here, we induced large, uniform, biaxial compressive strain (∼1.2%) by cooling, down to 10 K, single-layer WS_2_, MoS_2_, WSe_2_, and MoSe_2_ deposited on polycarbonate substrates. We observed a significant strain-induced modulation of neutral exciton energies, with blue shifts up to 160 meV, larger than in any previous experiments. Our results indicate a remarkably efficient transfer of compressive strain, demonstrated by gauge factor values exceeding previous results and approaching theoretical expectations. At low temperatures, we investigated the effect of compressive strain on the resonances associated with the formation of charged excitons. In WS_2_, a notable reduction of gauge factors for charged compared to neutral excitons suggests an increase in their binding energy, which likely results from the effects of strain added to the influence of the polymeric substrate.

## Introduction

Two-dimensional (2D) semiconductors possess unique electronic, optical, and optoelectronic properties. In particular, at the single-layer limit (1L), transition metal dichalcogenides (TMDs) exhibit a direct-bandgap electronic structure which is favorable for light emission.^1,2^ Additionally, the increased strength of Coulomb interactions at the single layer limit results in the formation of exciton bound states with large binding energies—up to hundreds of meV.^3,4^ This leads to robust excitonic light absorption and emission even at room temperature, making 2D-TMDs candidate materials for the design of ultrathin, flexible optoelectronic devices.^1,3,5−8^ Very recently, excitons in 2D semiconductors have been proposed to transmit information with low energy dissipation in the so-called excitonic devices.^9−11^ Furthermore, the manipulation of excitonic coherent states in single-layer TMDs has been proposed as a platform for quantum information technologies.^4,12−14^

In addition to their exceptional optical and mechanical properties, single-layer semiconductors exhibit a remarkable tunability in their electronic and optoelectronic properties.^15−17^ Strain has been widely recognized as a very effective tool for tuning the properties of 2D materials.^18−22^ For instance, strain modifies the electronic band structure of single- and few-layer TMD semiconductors, increasing or decreasing their band gap energy if the applied strain is either compressive or tensile.^21^ This, in turn, determines their optical spectra, modulating exciton energies.^22,23^ In fact, strain-induced changes in the electronic structure of TMDs have been extensively studied by tracking the shift in energy of excitonic emission or absorption using photoluminescence or reflectance spectroscopy measurements.^24−29^

Large amounts of uniform tensile strain, both uniaxial and biaxial, have been successfully induced in 2D semiconductors by bending them on flexible substrates.^6,7,24,27,30−32^ For instance, uniaxial strain up to ∼2.8% has been achieved in 1L-MoS_2_^24,30^ and gauge factors for excitons up to 100 meV/% have been demonstrated for biaxial strain.^27^ The effects of larger, nonuniform, biaxial tensile strain on the optical properties of TMDs have also been explored by nanoindentation methods^26^ and by pressurizing single-layer membranes.^29,33^ Some of these methods have also been applied to explore the impact of tensile strain on the binding energies and valley selectivity of excitonic species in single-layer TMDs.^34−36^ However, experimental methods to apply compressive strain and to study its effects on the properties of 2D materials are limited. Apart from hydrostatic pressure techniques,^28^ piezoelectric actuators have been recently employed to transfer modest amounts of purely biaxial compressive strain (∼0.3%).^37,38^ An alternative method for generating larger, homogeneous, biaxial compressive strain involves transferring it from substrates with large thermal expansion coefficients. This approach enables strain modulation through sample temperature control and has been recently applied to strain engineering of 2D materials.^39−43^ For instance, the effect of compressive strain in the photoresponse of MoS_2_ on polycarbonate has been investigated by cooling down to 80 K.^39^

In this work, we achieve large, reproducible amounts of uniform biaxial compressive strain, up to ca. −1.2% in single-layer TMDs. Strain is transferred from a polymer substrate, polycarbonate (PC), with a large thermal expansion coefficient during the cooling process from 300 to 10 K. To determine the effect of compressive strain, we compare the evolution of exciton resonances with temperature in single-layer TMD samples deposited on PC with similar ones on Si/SiO_2_ (substrate with negligible thermal expansion coefficient). When cooling down, we observe a significantly larger energy blue shift of the exciton resonances for samples on PC compared to samples on Si/SiO_2_ substrates, which we attribute to the strain transferred from the compression of the polymer substrate. From the comparison of exciton energy shifts for samples on both substrates, we determine strain gauge factors for neutral excitons in single-layer WS_2_, MoS_2_, WSe_2_, and MoSe_2_ under uniform biaxial compressive strain. Our results indicate an excellent transfer of strain to the single-layer TMDs, with exciton energy shifts and gauge factor values that substantially surpass the best results from previous compressive strain experiments. Furthermore, in the low-temperature regime, we reach a situation that combines high levels of biaxial compressive strain with enhanced spectroscopic resolution. This allows us to resolve additional resonances, which we associate with the formation of charged excitons (trions) in 1L-MoS_2_ and 1L-WS_2_. Strain gauge factors for trions are similar to those for neutral excitons in 1L-MoS_2,_ but smaller in 1L-WS_2_. This suggests an increase in the binding energy of trions with strain in 1L-WS_2_, which we discuss in terms of simplified models. We conclude that the large trion binding energy (∼80 meV) observed in strained 1L-WS_2_ likely originates from the combination of the substrate influence and the strain-induced changes in the electronic properties of the material.

## Results and Discussion

**Figure 1:**
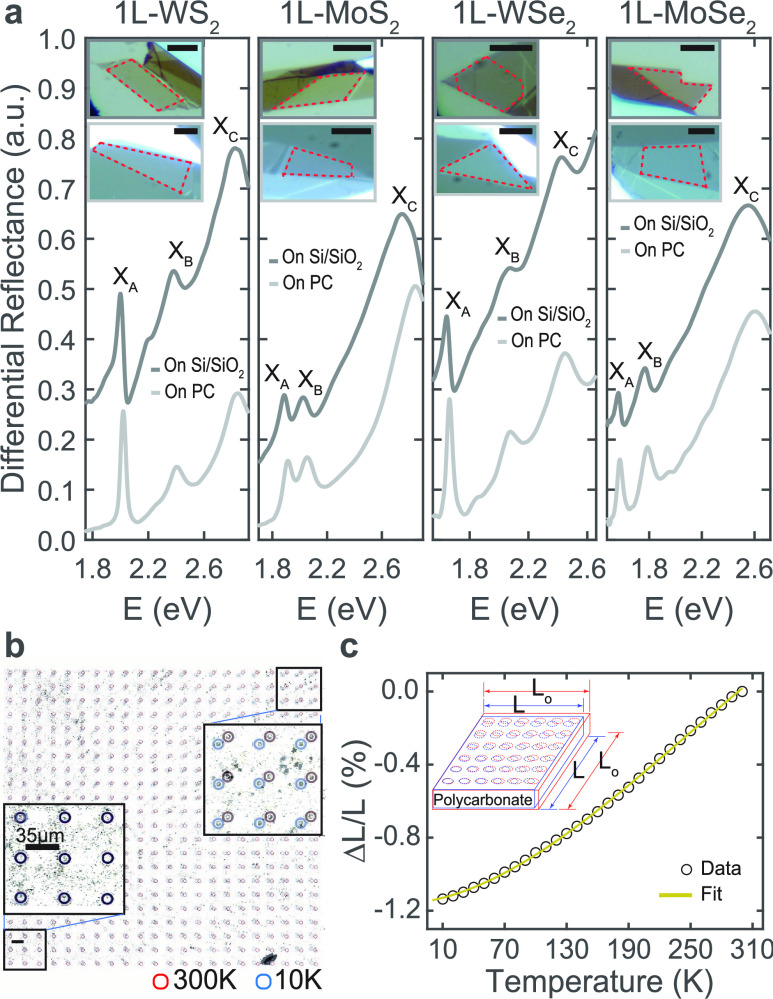
(a) Differential reflectance spectroscopy of single-layer samples of WS_2,_ WSe_2_, MoS_2_, and MoSe_2_ on Si/SiO_2_ (dark gray) and polycarbonate (light gray) substrates. The differential reflectance is calculated as (*R*_substrate_ – *R*_sample_)/*R*_substrate_ for samples on Si/SiO_2_ and as (*R*_sample_ – *R*_substrate_)/*R*_substrate_ for samples on PC, to enable a clear comparison between data obtained for samples on the two substrates. Data are shifted for clarity. The insets show optical images of the samples, where the single-layer area is marked with a dotted red line. Scale bars represent 10 μm. (b) Optical images of a resist micropillars grid (∼13 um of diameter) on a polycarbonate substrate at 300 and 10 K. The contrast of images has been adjusted to enhance the visibility of the micropillar positions. The perimeter of each micropillar is depicted in red for 300 K and in blue for 10 K.  (c) Average percentage decrease in the distance between pillars as the temperature is lowered, measured from images similar to those in (b) taken at 10 K intervals ranging from 300 to 10 K. The solid line represents a fitting of data to a polynomial function.

Single-layer samples of WS_2_, MoS_2_, WSe_2_, and MoSe_2_ were deposited on polycarbonate substrates (see ref (44) and the Materials and Methods section). Their thickness was confirmed by the energy position of exciton resonances in the differential reflectance spectra (Figure 1a), following ref (45), and by Raman spectroscopy (Supporting Information Section S1). Polycarbonate (PC) presents both a large Young modulus (*E* ∼ 2.5 GPa)^25^ and a large thermal expansion coefficient at room temperature (α ∼ 6.5 × 10^–5^ K^–1^),^39^ making it an ideal candidate substrate for strain transfer.^39,40^ To isolate the effect of strain from temperature, we prepared similar samples on Si/SiO_2_ substrates, a material with 2 orders of magnitude lower thermal expansion coefficient (α ∼ 5 × 10^–7^ K^–1^).^46^Figure 1a presents optical images and differential reflectance spectroscopy for single-layer samples of WS_2_, MoS_2_, WSe_2_, and MoSe_2_ transferred both on PC and on Si/SiO_2_ (the thickness of the SiO_2_ layer is 50 nm) substrates at room temperature under ambient conditions. The spectra exhibit distinct asymmetric resonances corresponding to three different species of excitons (*X*_A_, *X*_B_, *X*_C_). The different resonance shapes observed on the two substrates may stem from constructive interference effects at Si/SiO_2_, along with the broadening of the resonances on PC, which is likely the result of PC being comparatively less flat and homogeneous as a substrate than SiO_2_. An energy difference of ca. 10–20 meV is also observed between exciton resonances of the same 1L-TMD on Si/SiO_2_ and PC substrates. The dielectric constant of PC (ε_r_ ∼ 2.3–2.8^47,48^) being smaller than that of SiO_2_ (ε_r_ ∼ 3.9^49^), may result in different energy values for excitons in samples on PC compared to those on Si/SiO_2_. This variation stems from changes in the bandgap and exciton binding energies due to the different dielectric environments.^16,50^

Prior to studying the temperature-dependent optical properties of the 1L-TMD samples, the thermal compression of polycarbonate from 300 to 10 K was quantified by applying the method proposed in refs (39,40). Photoresist micropillars were patterned on top of a polycarbonate substrate (Figure 1b,c). The distance *L* between two pillars located at diagonally opposite corners of the image area in Figure 1b was tracked as a function of temperature. The thermal compression of the polycarbonate sample was determined as Δ*L*/*L* = (*L* – *L*_0_)/*L*_0_, where *L*_0_ is the distance in the uncompressed PC measured at room temperature. Δ*L*/*L* quantifies the compression level of the PC substrate and means the maximum strain that can be transferred to the 1L-TMDs on top of it. Compression increases down to 150 K and, below that, the compression rate decreases, reaching ∼1.2% at 10 K, in agreement with ref (51). A fit to a polynomial expression will be used to correlate substrate compression and temperature .

**Figure 2:**
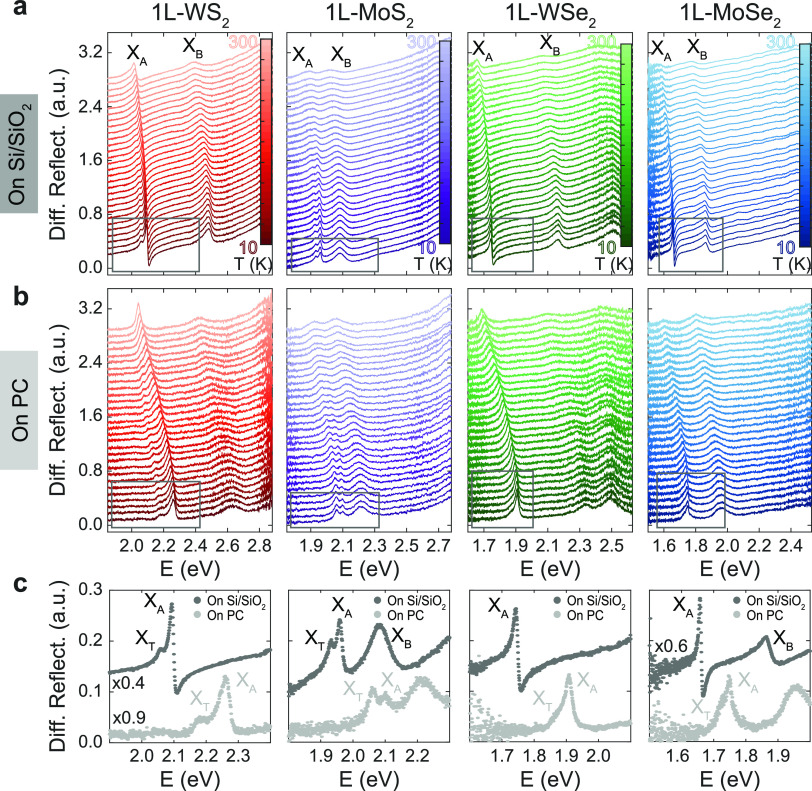
(a, b) Differential reflectance as a function of temperature for single-layer TMD samples on (a) Si/SiO_2_ and (b) polycarbonate (PC) substrates. Data corresponding to each single-layer material (WS_2_, MoS_2_, WSe_2_, and MoSe_2_) are accordingly labeled at the top row of the figure. Data are vertically shifted for clarity. (c) Comparison of differential reflectance spectra obtained at base temperature (10 K) for samples on Si/SiO_2_ and PC substrates, respectively, zooming into the *X*_A_ resonance energy range, which is marked with a gray line rectangle in (a) and (b).

Next, we compare variable-temperature micro-reflectance measurements on TMD single layers on PC and on Si/SiO_2_—substrate with negligible thermal compression coefficient (see the Materials and Methods section and Supporting Information Section S2). The temperature evolution of the differential reflectance is presented in Figure 2a for single layers of four different TMDs (1L-WS_2_, 1L-MoS_2_, 1L-WSe_2_, and 1L-MoSe_2_). At room temperature, two resonances are visible in all four materials, corresponding to the A and B excitons, labeled *X*_A_ and *X*_B_ in Figure 2a. These spectra are taken under vacuum conditions (*P* < 10^–6^ mbar) and an energy shift of ∼20 meV in the energy positions of *X*_A_ and *X*_B_ is observed when compared to the ambient spectra in Figure 1. This shift is likely due to the absence of adsorbents that affect the dielectric environment.^15^

As temperature decreased, on Si/SiO_2_ substrates, the exciton peaks of all four single-layer TMDs shifted toward higher energies. This trend follows the expected increase of the bandgap energy due to the freezing of electron–phonon interactions and the slight change in bonding lengths.^52,53^ On Si/SiO_2_ substrates, we attribute this shift to the sole effects of temperature, considering substrate deformation negligible. Notably, for samples on PC substrates, a significantly larger blue shift of *X*_A_ and *X*_B_ peaks with decreasing *T* was observed compared to samples on Si/SiO_2_. We attribute the extra energy shift observed for samples on PC to the strain transferred to the 1L-TMDs from the thermal compression of the PC substrate.^40^ At low temperatures, as the width of the resonances decreases, a peak can be resolved at energies lower than those of exciton *A*. This peak is more pronounced in the 1L-MoS_2_ and 1L-WS_2_ spectra for both Si/SiO_2_ and PC substrates (Figure 2c), where it clearly appears below 200 K. A shoulder on the left side of *X*_A_ also appears for 1L-WSe_2_ and 1L-MoSe_2_, but only in samples on PC and at the base temperature (Figure 2c). In previous works, a similar peak has been observed in the optical spectra of various 1L-TMDs and has been typically associated with the absorption or emission from a negatively charged exciton, also known as a trion.^54−57^ Given its resemblance to trionic resonances, we label this resonance *X*_T_ in Figure 2c.

**Figure 3:**
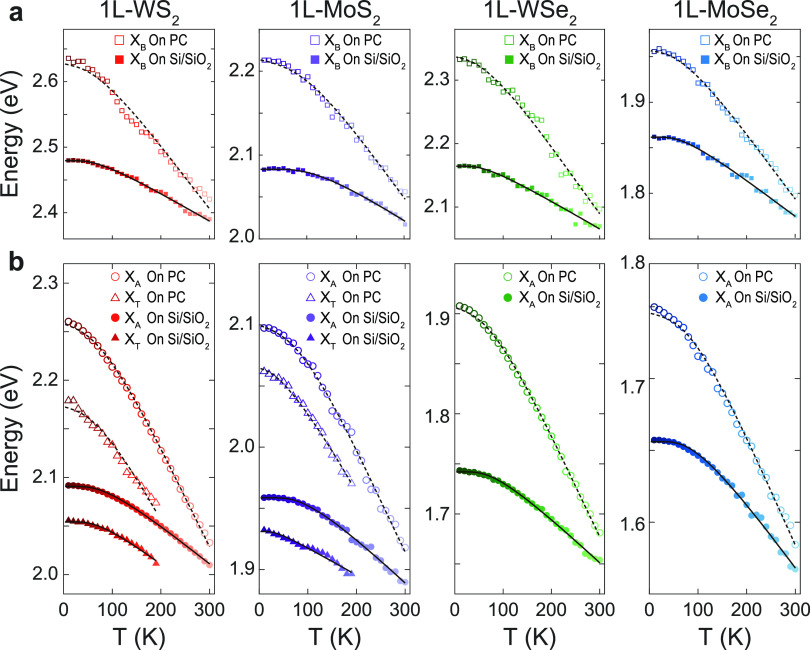
(a, b) Data points representing the energy positions for resonances (a) *X*_B_ and (b) *X*_A_ and *X*_T_ extracted from data in Figure 2 for 1L-WS_2_, 1L-MoS_2_, 1L-WSe_2_, and 1L-MoSe_2_, on Si/SiO_2_ and PC substrates, respectively. In the case of data on Si/SiO_2_ substrates, solid lines represent the fit of data to the model for the bandgap energy evolution with temperature proposed in ref (52). In the case of samples on PC substrates, the dashed line represents the exciton energy evolution estimated from the addition of the effect of strain (calculated later in the article from the analysis in Figure 4) to the effect of temperature. The effects of temperature are estimated from the fitting to the bandgap model for data in the same material on a Si/SiO_2_ substrate.

The temperature evolution of the energy position of resonances *X*_A_, *X*_B_, and *X*_T_ (see Supporting Information Section S3 for details) is presented in Figure 3. For all four 1L-TMDs on Si/SiO_2_ substrates, *X*_A_ and *X*_B_ energy positions can be fitted to a model that describes the temperature dependence of the bandgap energy^52^ (solid lines in Figure 3, Supporting Information Section S4). For both 1L-MoS_2_ and 1L-WS_2_ on Si/SiO_2_ substrates, *X*_T_ follows a trend similar to that of *X*_A_ (see Figure 3b) and fitting with the same bandgap model yields similar parameters as for *X*_A_ and *X*_B_ (see Supporting Information Section S4). This fact suggests that the blue shift of exciton energies—which are the difference between bandgap energy and exciton binding energy—is dominated by changes in the bandgap and that temperature appears to have no significant effect on the binding energies of the corresponding excitonic states. The effect of temperature-induced changes in the substrate dielectric screening can be also disregarded. According to ref (48), the dielectric constant of polycarbonate decreases ∼2% at cryogenic temperature. This variation may lead to changes of just a few meV in the exciton energies of 1L-TMDs.^16,50^

**Figure 4:**
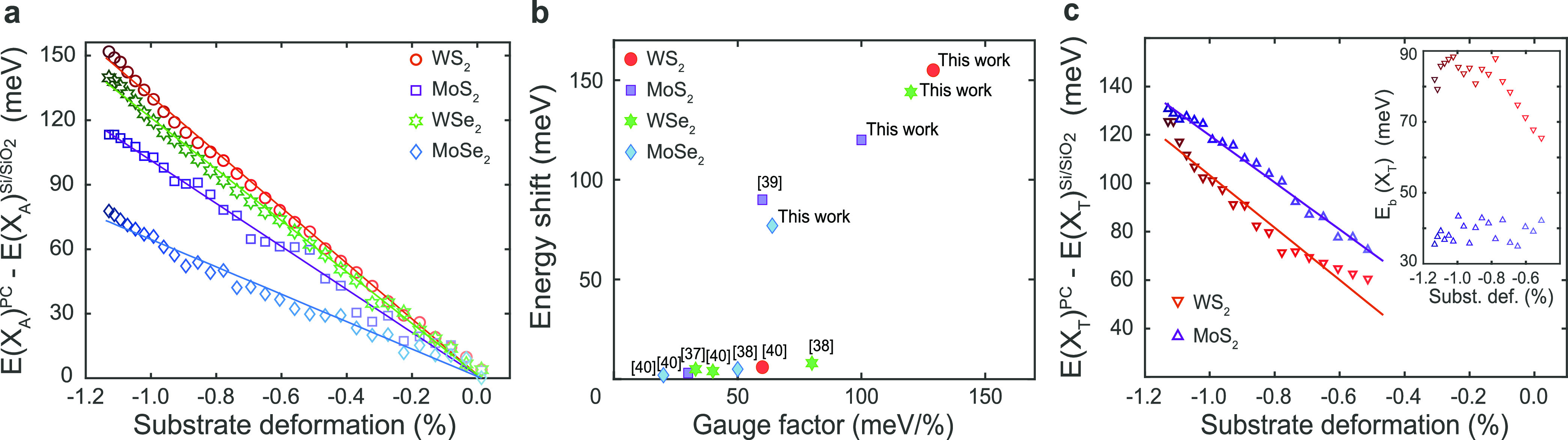
(a, c) Data points represent the energy difference Δ*E*(*X*) = *E*_X_^PC^ – *E*_X_^Si/SiO_2_^ between (a) *X*_A_ excitons and (c) *X*_T_ charged excitons on the two different substrates, PC and Si/SiO_2_, as a function of substrate deformation (estimated as Δ*L*/*L* in Figure 1c), for a single layer of each of the four materials under study. In (a), the exciton energy difference between substrates at room temperature has been subtracted from the data for clarity (see Supporting Information Section S6). Solid lines represent linear fits to the data. The slope of these linear fits yields the gauge factors presented in Table 1. (b) Comparison of the maximum energy blueshift of the bandgap energy and gauge factor values obtained in this work with those from other works on biaxial compressive strain.^37−40^ The bandgap energy shift is assumed to be the same as for *X*_A_ when it applies. For a fair comparison, the effect of temperature has been removed in other thermal compression experiments using the same method applied to our data. The inset in (c) shows the energy difference between *X*_T_ and *X*_A_, associated with the binding energy of a negatively charged exciton, *E*_b_(*X*_T_) = *E*(*X*_T_) – *E*(*X*_A_), for 1L-MoS_2_ and 1L-WS_2_ on PC.

To separate the effects of strain and temperature, we compared the energy positions of exciton peaks on PC (*E*_X_^PC^) and the corresponding values for similar samples on Si/SiO_2_ (*E*_X_^Si/SiO_2_^). The energy difference between each resonance on the two substrates, Δ*E*(*X*) = *E*_X_^PC^ – *E*_X_^Si/SiO_2_^, is presented as a function of substrate deformation in Figure 4 (as a function of *T* in Supporting Information Section S5). Assuming that Si/SiO_2_ data compiles the effects of temperature, Δ*E*(*X*) represents the blue shift in the exciton energy owing to the strain transferred from the compression of the PC substrate. Indeed, we find that Δ*E*(*X*) follows an almost linear relationship with substrate deformation and thus, with the amount of transferred strain (Figure 4a), in agreement with previous experimental and theoretical works.^22,40,58^ Fitting Δ*E*(*X*) versus substrate deformation to a linear expression (Figure 4a) yields experimental strain gauge factors—defined as the exciton energy shift due to 1% of substrate deformation—for excitons A and B, which are summarized in Table 1 (see Supporting Information Section S6 for more details). The uncertainty values presented in Table 1 are the statistical errors obtained from the linear regression of the data in Figure 4. Gauge factors obtained for different samples differ by up to ∼10 meV/% (see Supporting Information Section S7). The extracted gauge factor values for *X*_A_ follow the hierarchy WS_2_ > WSe_2_ > MoS_2_ > MoSe_2_, in agreement with refs (22,40). This hierarchy has been attributed to the specific effects of strain on the band structure of each material.^40^

**Table 1:** Strain Gauge Factors Obtained from the Slope of Exciton Energies versus Substrate Deformation in Figure 4^a^

Gauge factor (meV/%)	*X*_A_ this work	*X*_A_ theory	*X*_B_ this work	*X*_B_ theory	*X*_T_ this work
1L-WS_2_	–129 ± 3	–151^40^	–112 ± 10	–130^40^	–108 ± 13
		–144.0^22^		–123.8^22^	
1L-MoS_2_	–100 ± 3	–110^40^	–90 ± 5	–107^40^	–97 ± 3
		–112.5^22^		–109.8^22^	
1L-WSe_2_	–120 ± 3	–134^40^	–129 ± 10	–111^40^	
		–131.2^22^		–109.7^22^	
1L-MoSe_2_	–64 ± 4	–90^40^	–66 ± 4	–89^40^	
		–98.6^22^		–97.2^22^	

a Results are compared to theoretical predictions from refs (22,40).

It is important to note that experimental gauge factors are calculated for substrate deformation and not actual strain. Therefore, a larger value of the experimental gauge factor, a larger energy shift for a given amount of substrate deformation, implies a more efficient transfer or strain. In Figure 4b, we compare our results to previous works on compressive strain in 1L-TMDs, considering two figures of merit: experimental gauge factor values, which assess the efficiency of the strain transfer, and the maximum induced energy shift, proportional to the maximum degree of compressive strain achieved. The large gauge factor values obtained for *X*_A_ in our work point to an excellent transfer of compressive strain from PC substrates to 1L-TMDs. This efficient transfer of strain, together with the level of deformation achieved, results in larger modulations of exciton energy for all four materials than in any previous compressive strain experiments. On the one hand, our gauge factor values are of similar magnitude to those obtained from piezo-actuator experiments,^37,38^ but the amount of deformation achieved in our experiments is an order of magnitude larger. On the other hand, we obtain a significantly more efficient transfer of strain than in previous thermal compression experiments, likely due to performing the experiments under vacuum and maintaining precise control of temperature change rates (see the Materials and Methods section). In fact, our gauge factor values are nearly twice as high as those reported in previous experiments involving thermally induced strain.^39,40^ Furthermore, our gauge factor values are comparable to the best values achieved for biaxial tensile strain using bending techniques,^27,31,32^ or pressurized membranes^23,29^ (see Supporting Information Section S8 for a detailed comparison). Moreover, these values closely align with those predicted by ab initio calculations^22,40^ (see Table 1). Gauge factor values obtained from different computational methods exhibit variations of up to 10 meV/%. Theoretical gauge factor values, calculated as the energy shift per % of actual strain, can be considered an upper bound for experimental results. Differences in the quantitative agreement between theoretical and experimental values for different materials likely arise from the addition of experimental and theoretical uncertainties.

To assess the validity of our approach in separating the effects of temperature and strain, we plot, with the dashed line in Figure 2, the result of adding the temperature evolution of exciton energies for Si/SiO_2_ substrates (assumed as negligible strain) plus a linear term on strain with the gauge factor as the slope coefficient. The good agreement with the experimental data suggests that the sole effect of temperature is similar for samples on both substrates and that disregarding changes in substrate effects with temperature is a reasonable assumption. Gauge factors for *X*_B_ are obtained from a similar analysis (see Supporting Information Section S6) and compared in Table 1 with the corresponding theoretical estimates. In general, gauge factor values obtained for *X*_B_ are, within a larger error range, similar to those obtained for *X*_A_.

Finally, we focus on the evolution of *X*_T_ resonances with substrate deformation for the two materials, MoS_2_ and WS_2_, where this peak can be resolved. On Si/SiO_2_ substrates, *X*_T_ undergoes a similar energy shift with temperature as *X*_A_ in both 1L-WS_2_ and 1L-MoS_2_ (Figures 2a and 3b; Supporting Information Section S4). On PC substrates, *X*_T_ in 1L-MoS_2_ exhibits a similar energy blue shift as X_A_ (Figures 2b and 3a). However, in 1L-WS_2_ on PC, *X*_T_ deviates from this trend and displays a significantly smaller blue shift compared to *X*_A_ as temperature decreases (Figures 2b and 3a). To quantify this observation, we estimate gauge factors for *X*_T_ using the same method applied previously for *X*_A_ (Figures 3b and 4c). For 1L-MoS_2_ on PC, we obtain an *X*_T_ gauge factor that is approximately the same, within error, as that for *X*_A_. However, the gauge factor obtained for *X*_T_ in the case of 1L-WS_2_ on PC is ∼20 meV/% smaller compared to *X*_A_. Before addressing the potential causes of this effect, we discuss the origin of the resonance *X*_T_. For samples on Si/SiO_2_ substrates, the resemblance of resonances with those in other works^54,56^ suggests that *X*_T_ arises from trion formation. Moreover, the relative intensity of *X*_T_ versus *X*_A_ being higher for 1L-MoS_2_ and 1L-WS_2_ on PC than on Si/SiO_2_ (see Supporting Information Section S3) is also compatible with a trion origin for *X*_T_, since trion relative spectral weight is highly dependent on free-carrier density.^59^ Single-layer MoS_2_ and WS_2_ are typically described as intrinsically n-type doped semiconductors, and their doping level is often altered by charge transfer from the substrate. An alternative origin for *X*_T_ from a strain-induced direct-to-indirect bandgap transition (see refs (22,23)) can be ruled out. First, if *X*_T_ originated from the indirect gap, it would be expected to appear similarly in 1L-WSe_2_ since the evolution of the band structure with strain is nearly identical for single-layer WSe_2_ and WS_2_.^22^ Second, to further understand the origin of *X*_T_, we performed similar experiments in 1L-WS_2_ deposited on a different polymer–polypropylene (PP) substrate. For 1L-WS_2_ on PP, we obtained *X*_A_ gauge factors that were close to those obtained on PC (see Supporting Information Section S9), indicating a comparable strain transfer. Interestingly, the *X*_T_ peak observed on PC was absent for 1L-WS_2_ on PP. Since the intensity and energy position of trion resonances strongly depend on the doping level of 1L-TMDs, they may significantly differ from substrate to substrate.^56,57,60^ Therefore, the observed changes for *X*_T_ between samples on different substrates point to its origin in the formation of a charged quasiparticle, ruling out neutral excitons and biexcitons as potential sources.

All of the above support a trionic origin for *X*_T_ in 1L-MoS_2_ and 1L-WS_2_. This could also be the origin of the *X*_T_ shoulder observed in 1L-WSe2 and 1L-MoSe2 spectra at base temperature (Figure 2c), which could also stem from a higher doping level for these materials on PC compared to Si/SiO_2_. However, in 1L-WSe_2_ and 1L-MoSe_2_, *X*_T_ resonances cannot be resolved at higher temperatures, limiting the study of their evolution with strain. We therefore focus on the investigation of strain effects for charged excitons in 1L-MoS_2_ and 1L-WS_2_. The energy difference between *X*_A_ and *X*_T_, *E*_b_^T^ = *E*(*X*_A_) – *E*(*X*_T_), represents the binding energy of trions. This quantity remains approximately constant with temperature for both 1L-MoS_2_ and 1L-WS_2_ on Si/SiO_2_, around 30 and 40 meV, respectively (Supporting Information Section S10). On PC, *E*_b_^T^ also remains constant within 35–45 meV for 1L-MoS_2_. However, for 1L-WS_2_ on PC, *E*_b_^T^ is significantly larger and seems to increase with compressive strain, reaching ∼80 meV (see the inset of Figure 4c). The effect of strain on the binding energies of neutral excitons has been both experimentally and theoretically explored, demonstrating changes of the order of 10 meV/%.^22,35,58,60^ However, the effect of strain on the binding energies of trions has been less studied and only in the case of tensile strain experiments.^26,32^

In a first approximation, the binding energies of excitons for an intrinsic 2D semiconductor depend solely on the exciton-reduced mass μ and the screening length ρ for the Coulomb interactions within the material,^4,61−63^ which are expected to change under applied strain.^58,64^ From first-principles calculations (see the Materials and Methods section), we estimated that a 1.5% biaxial compressive strain can result in an ∼10% increase in the reduced mass for both 1L-MoS_2_ and 1L-WS_2_ (Supporting Information Section S11). Based on the results in ref (65), we estimated a decrease of ∼4 and ∼2% in the screening lengths of 1L-MoS_2_ and 1L-WS_2_, respectively, under −1.5% strain. Different models for calculating trion binding energies (see refs (61−63)) suggest that increasing μ and decreasing ρ both contribute to an increase in the binding energies of negative trions. However, the estimated increase due to ca. −1.5% strain is found to be modest, less than 3 meV, for both 1L-WS_2_ and 1L-MoS_2_ (from refs (61,63); see Supporting Information Section S12). Even considering the combined effect of strain and the different dielectric screening from SiO_2_ and PC substrates, *E*_b_^T^ would increase only by as much as ca. 6–7 meV for both 1L-MoS_2_ and 1L-WS_2_ on PC with respect to Si/SiO_2_ at low temperatures (see Supporting Information Section S10). Therefore, considering the sole effect of strain on the intrinsic electronic structure may not suffice to explain the observed large *E*_b_^T^. It is important to note that trion binding energies are strongly influenced by carrier concentration,^56,57,60^ which can vary substantially between different samples due to charge transfer between the substrate and each specific material. Differences in the intensity ratios between *X*_T_ and *X*_A_ suggest an increase in electron concentration for samples on PC in comparison to samples on Si/SiO_2_. A higher electron concentration can explain the overall increased binding energy for trions in both 1L-MoS_2_ and 1L-WS_2_ on PC compared to the same materials on Si/SiO_2_. However, in 1L-WS_2_, *E*_b_^T^ appears to increase further with induced strain. Interestingly, various works suggest that the doping level of TMDs may change with strain.^66,67^ Additionally, a large energy shift for WS_2_ trions has been observed under inhomogeneous tensile strain at low temperatures in ref (26), which could arise from strain-induced changes in carrier concentration. In summary, we propose that the further increase in *E*_b_^T^ for trions with biaxial compressive strain in 1L-WS_2_ may arise from a combination of factors, including steady-state substrate effects as well as dynamic strain-induced changes in the intrinsic electronic structure and in the free-carrier concentration of the material. Doping level variations modify the dielectric landscape and result in a renormalization of energy levels, leading to dramatic changes in the overall optical spectrum of the material, and for trion energies, in particular. Therefore, a further understanding of the observed behavior of *X*_T_ resonances with strain in 1L-WS_2_ requires more sophisticated models than those currently available, capable of capturing the exact interplay of strain and doping and their impact on the excitonic properties of the material.

## Conclusions

In conclusion, our findings demonstrate an excellent transfer of uniform, biaxial compressive strain (ca. −1.2%) to single-layer materials from the thermal compression of polymer substrates down to 10 K, demonstrated by the large gauge factor values obtained. This strain induces a remarkably large modulation in the exciton energies in single-layer TMDs (up to 160 meV), larger than values obtained in previous compressive strain experiments. The analysis of data over a large temperature range allows us to precisely separate the effects of strain from those of temperature in exciton energies. Furthermore, at low temperatures, we resolve further resonances that we associate with trionic states and study their behavior under biaxial compressive strain. We found a substantial increase in the trion binding energy of 1L-WS_2_ on PC at 10 K, which may arise from a combination of substrate-related effects and strain-induced changes in the electronic properties of the material. These findings go beyond the scope of existing models and may require further investigation. The large modulation of exciton resonances in our results holds relevance for applications that require precise manipulation of excitonic states in 2D semiconductors, such as valley selectivity or exciton transport. Moreover, this work demonstrates a simple method to explore the effect of large, uniform compressive strain on low-temperature phenomena in other 2D materials.

## Materials and Methods

### Fabrication of Single-Layer TMD Samples

MoS_2_, WS_2_, MoSe_2_, and WSe_2_ crystals were mechanically exfoliated onto transparent poly(dimethylsiloxane) (gel film from Gel-Pak) substrates for inspection under an optical Motic BA310 metallurgical microscope. From differential reflectance measurements at room temperature, we were able to identify single-layer TMD flakes. Selected flakes were transferred by a dry-transfer method^44^ onto either a 250 μm thick polycarbonate film with size of 6 mm × 6 mm or a 6 mm × 6 mm Si/SiO_2_ substrate with a 50 nm oxide layer.

### Variable-Temperature Differential Reflectance Measurements

Differential reflectance measurements were performed with a homemade microscope setup based on ref (45). Samples were illuminated by a white light source that was focused on the sample by an infinite corrected 50× objective. Because of the different energy positions of exciton resonances for different materials, two different light sources were used: a SOLIS-3C lamp (400–900 nm) from Thorlabs was used to illuminate the sulfide samples, whereas a halogen lamp (500–1100 nm) from a Motic BA310 microscope was used for selenide samples. A diaphragm is used to reduce the illumination area to a few tens of micrometers on the sample around the measurement spot. A tube lens of focal length *f* = 20 cm collects the reflected light into an optical fiber. The fiber core (105 μm diameter) determines the diameter of the spot at the sample from which light is collected (∼2 μm). The fiber guides the reflected light to a CCS200/M compact spectrometer (Thorlabs). The acquired spectra from the sample and from the substrate are used to calculate the differential reflectance as *DR* = (*R*_sample_ – *R*_substrate_)/*R*_substrate_, where *R*_sample_ and *R*_substrate_ are the reflected light intensities at the sample and substrate, respectively.

The home-built microscope setup described above is located at room temperature above the window of a table-top closed-cycle cryostat (AttoDry 800, Attocube Gmbh) that allows to control the temperature of the sample from 10 to 300 K. The cryostat is equipped with a piezoelectric controller to position the sample under the microscope objective. Reflectance spectroscopy measurements were recorded for both cooling and heating of the samples over a range from 300 to 10 K (at a rate of ∼1 K/min) under cryogenic vacuum (<1 × 10^–6^ mbar) for the same locations on sample and substrate. Optical images of the samples during the cooldown or warmup process (see Supporting Information Section S2) allow us to perform spectroscopy at the same location of the sample and substrate at different temperatures. Optical images are inspected to check that no wrinkles or additional defects appear in the single-layer flakes with lowering temperature, suggesting a smooth transfer of strain (see Supporting Information Section S2).

### DFT Calculations and Determination of Reduced Masses with Applied Strain

The effective masses are obtained from the electronic structure calculations performed within the density functional theory formalism using the plane-wave pseudopotentials method, as implemented in the pwscf code of the QUANTUM ESPRESSO package.^68^ The exchange and correlation potentials were treated within the revised Perdew–Burke–Ernzerhof generalized gradient approximation (PBEsol).^69^ Fully relativistic pseudopotentials were considered to include spin–orbit coupling. For all elements, we employed the ultrasoft pseudopotentials obtained from the PS library.^70^ We used a Monkhorst–Pack grid of 30 × 30 × 1 points, and the energy cutoff for the plane-wave expansion was 110 Ry. In order to avoid interaction between the periodic images, we introduced a vacuum region of 16 Å. We relax the atomic positions for each strain while keeping the unit cell constant with the BFGS quasi-newton algorithm with a convergence threshold on forces of 1 × 10^–3^ Ry/a_0_.

We computed the effective masses at the *K* point for the two high-symmetry directions *K*Γ and *KM* of the Brillouin zone, and the averaged value between the two directions is shown in Figure S9. We used two different methods to compute numerically the second derivative of the energy band dispersion. First, we performed a quadratic fit using the three nearest points to the *K* point spaced by 0.01 Å^–1^ for each direction. Second, we performed a third-order spline interpolation of the same points. The results of both methods were nearly identical, with less than 1% of the difference.

## Supporting Information Available

Further experimental characterization of samples (Raman spectroscopy and optical images), further details on data analysis (parameters from data fittings, resonance position, and intensity determination), gauge factor values for additional samples, a bibliographic comparison of gauge factor values for 1L-TMDs, and DFT estimations of effective masses with strain and binding energy estimations as a function of strain (PDF)
