# Supplementary material for: Large Biaxial Compressive Strain Tuning of Neutral and Charged Excitons in Single-Layer Transition Metal Dichalcogenides
Source: ACS Appl Mater Interfaces. 2023 Nov 30;15(49):57369–78. doi: 10.1021/acsami.3c13281 (PMC10726316; doi:10.1021/acsami.3c13281)
Supplement: Supplementary file 1 — am3c13281_si_001.pdf [file am3c13281_si_001.pdf]

Supporting Information for

"Large Biaxial Compressive Strain-Tuning of Neutral and Charged Excitons in Single-Layer Transition-Metal-Dichalcogenides"

Eudomar Henríquez-Guerra[1,2], Hao Li[3], Pablo Pasqués-Gramage[1], Daniel Gosálbez-Martínez[1,2], Roberto D'Agosta[4,5], Andres Castellanos-Gomez[3,], M. Reyes Calvo[1,2,*]*

[1] Departamento de Física Aplicada, Universidad de Alicante, 03690 Alicante, Spain

[2] Instituto Universitario de Materiales IUMA, Universidad de Alicante, 03690, Alicante, Spain

[3] Materials Science Factory, Instituto de Ciencia de Materiales de Madrid, Consejo Superior de Investigaciones Científicas, 28049, Madrid, Spain

[4] ETSF Scientific Development Center, Departamento de Fısica de Materiales, Universidad del País Vasco, E-20018 San Sebastián, Spain

[5] IKERBASQUE, Basque Foundation for Science, E-48013 Bilbao, Spain

*E-mail: andres.castellanos@csic.es, reyes.calvo@ua.es

Contents

S1. Raman spectroscopy of 1L-TMD samples (pages 2-3)

S2. Optical images of samples (page 4)

S3. Determination of energy positions and intensity of exciton resonances from fitting to Aspnes equation (pages 5-8)

S4. Parameters from fitting exciton energies in 1L-TMDs on Si/SiO_2 to the O'Donell model for bandgap evolution with temperature (page 9)

S5. E_X^{PC} - $E_X^{Si/SiO2}$ as a function of temperature (page 10)

S6. Linear fitting analysis of $E(X)^{PC}$- $E(X)^{Si/SiO2}$ with substrate deformation for X_A, X_B and X_T (pages 11, 12)

S7. Gauge factors for additional 1L-TMDs flakes (page 13)

S8. A comparative of strain gauge factors for excitons in 1L-TMDs (pages 14-15)

S9. Differential reflectance for 1L-WS_2 on a Polypropylene substrate (pages (16-17)

S10. Trion binding energies as a function of temperature for 1L-MoS_2 and 1L-WS_2 (page 18)

S11. Exciton reduced mass as a function of strain from DFT calculations (pages 19-20)

S12. Trion binding energies estimations (pages 21-22)

Supporting References (pages 23-24)

S1. Raman spectroscopy of 1L-TMD samples

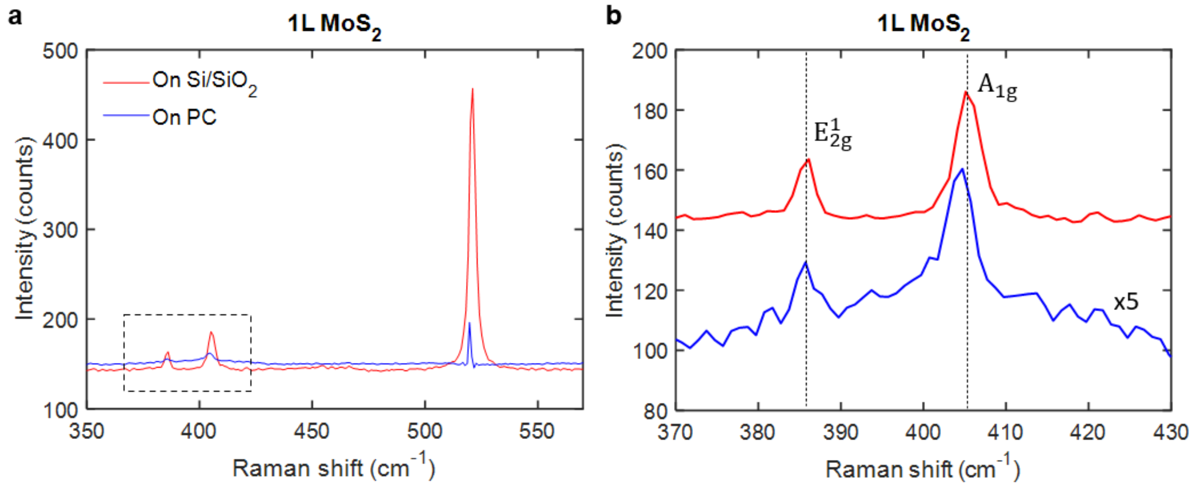

Figure S1. Raman spectra of single-layer MoS_2 deposited on a Si/SiO_2 substrate (red line) and on polycarbonate (PC) (blue line). The spectra were acquired at room temperature using a commercial Raman microscope (Jasco NRS-5100) with an excitation wavelength of 532 nm. The laser spot had a diameter of approximately 1.5 µm, and the total power was 400 µW. (a) Full spectrum. (b) Zoomed-in view of the dashed square region in panel (a). The dashed lines in panel (b) indicate the positions of the peaks corresponding to the E^1_{2g} and A_{1g} modes for 1L-MoS_2 deposited on Si/SiO_2. The spectrum represented with a blue line in panel (b) has been vertically shifted for better comparison.

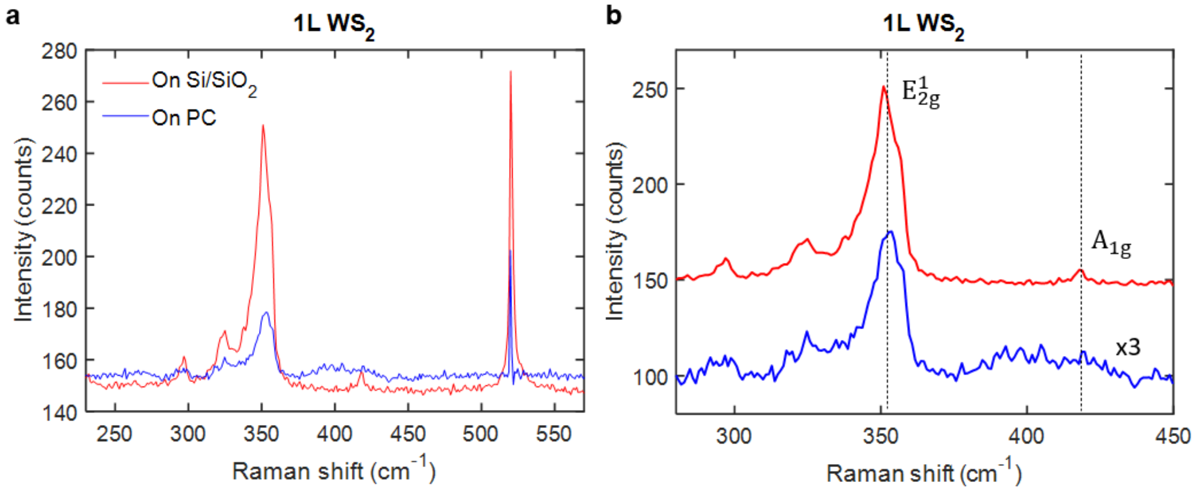

Figure S2. Raman spectra of single layer WS_2 deposited on Si/SiO_2 (red line) and on polycarbonate (PC) (blue line). The spectra were acquired at room temperature using a commercial Raman microscope (Jasco NRS-5100) with an excitation wavelength of 532 nm. The laser spot had a diameter of approximately 1.5 µm, and the total power was 400 µW. (a) Full spectrum (b) Zoomed-in view of the region of interest around the E^1_{2g} peak in (a). The dashed lines in panel (b) indicate the position of the peaks corresponding to the E^1_{2g} and A_{1g} mode positions for 1L-WS_2 deposited on Si/SiO_2. The spectra represented with a blue line in (b) has been vertically shifted for better comparison.

Table S1. Raman shift difference between the A_{1g} and E^1_{2g} Raman modes for 1L-MoS_2 and 1L-WS_2

**1L MoS_2**		**1L WS_2**	
Substrate	(A_{1g} - E^1_{2g}) (cm^{-1})	Substrate	(A_{1g} - E^1_{2g}) (cm^{-1})
Silicon oxide	19.7 ± 0.5	Silicon oxide	66.6 ± 0.5
Polycarbonate	18.5 ± 0.5	Polycarbonate	63.4 ± 0.5
Silicon oxide (ref. [s1])	19-20	Silicon oxide (ref. [s2])	65.8

The Raman shift difference between the A_{1g} and the E^1_{2g} peaks clearly indicates the single layer character of MoS_2 and WS_2 samples deposited on both Si/SiO_2 and PC substrates. Similarly, it indicates that the sample of WS_2 on Si/SiO_2 is also a single layer.

S2. Optical images of samples at different temperatures

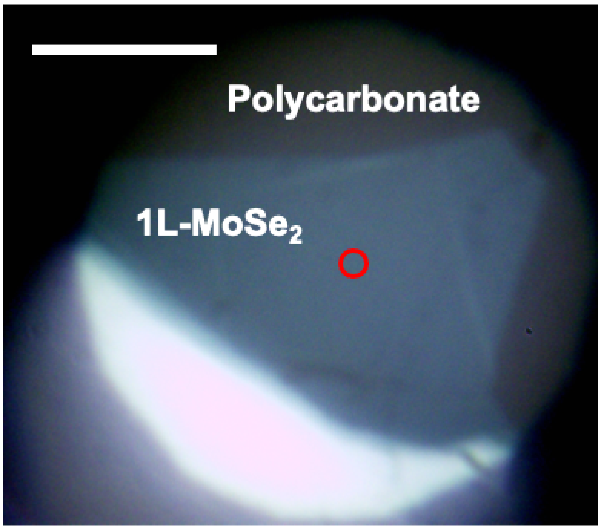

Figure S3. Optical image of 1L-TMD flake acquired during micro-reflectance measurements. The red circle marks the spot from which light is collected during spectroscopy measurements. Scale bar represents 20μm.

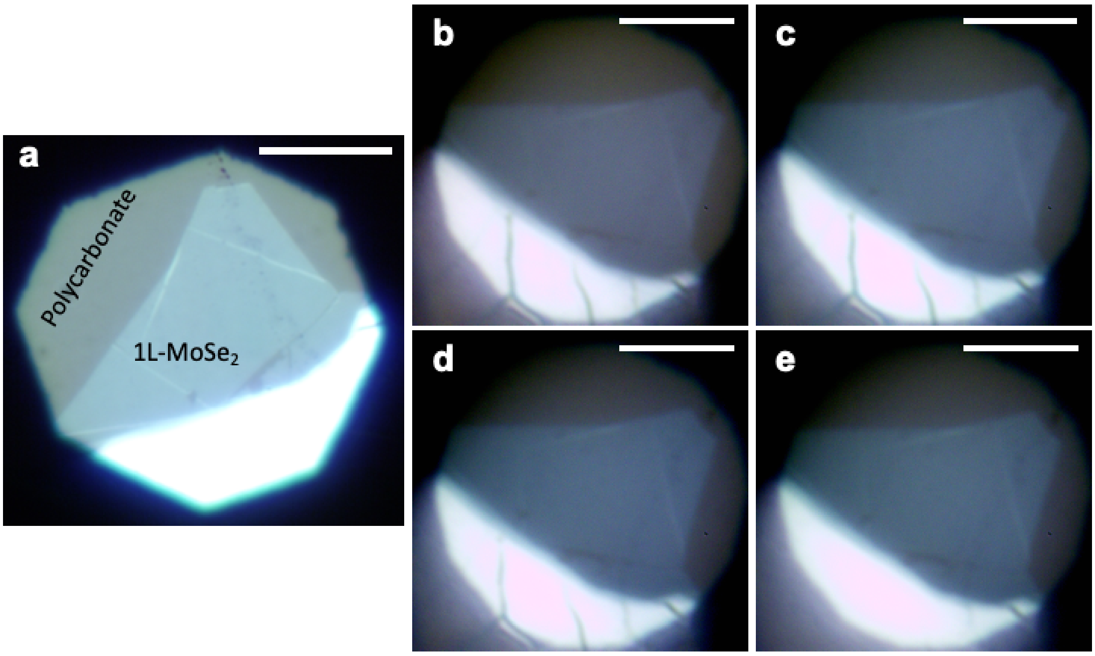

Figure S4. Optical images of 1L-$MoSe_2$ flake a) before cool-down, taken using a modified optical microscope in ambient conditions. b-e) Optical images of the same flake taken with the home-made setup described in the Methods section with the sample inside the cryostat: b) at base temperature (10 K), and c-e) during a warm-up cycle at c) 100 K, d) 200 K, e) 300K. Scale bar in (a-e) represents 20μm.

S3. Determination of energy position and intensity of exciton resonances from analysis of differential reflectance data

The position of the resonances corresponding to different excitons was determined by applying the first and second derivative criteria. In this regard, the position of the local maxima associated with each exciton was obtained from the energy value where the first derivative of the reflectance spectrum becomes zero and its second derivative is negative (see Fig. S5). This method was used to extract the position of resonances X_A and X_B for the four materials.

Strictly, for Si/SiO_2 data, this method determines the position of the left positive peak rather than the center of the resonance. However, we estimate the error introduced because of this to be below 5 meV and therefore can be disregarded. This method provides more accurately the evolution of the resonance positions over the whole range of temperatures than alternative methods such as the resonance fitting method proposed below, which is advantageous instead in the low temperature regime.

Also, the determination by the derivative analysis proved imprecise for determining the position of the X_T resonances, due to their less prominent peak. In this case, a fit to a sum of resonances X_A and X_T, each of one described by the Aspnes equation [s3], was used to corroborate the positions found by the derivative criterion (see Fig. S6). The Aspnes formula is given by:

$$\frac{\Delta R}{R}(E) = Re\left[\sum_{j=1}^{n} C_j e^{i\theta_j}\left(E - E_j - i\Gamma_j\right)^{-m_j}\right] \quad \text{(Eq. S1)}$$

where each optical resonance *j* is characterized by its amplitude (C_j), phase (θ_j), and broadening (Γ_j). The energy position of the resonance is denoted by E_j. The parameter (*m*) in the equation is associated with the type of optical transition, and specifically for the excitonic transition, its value is equal to 2, according to Ref. [s4].

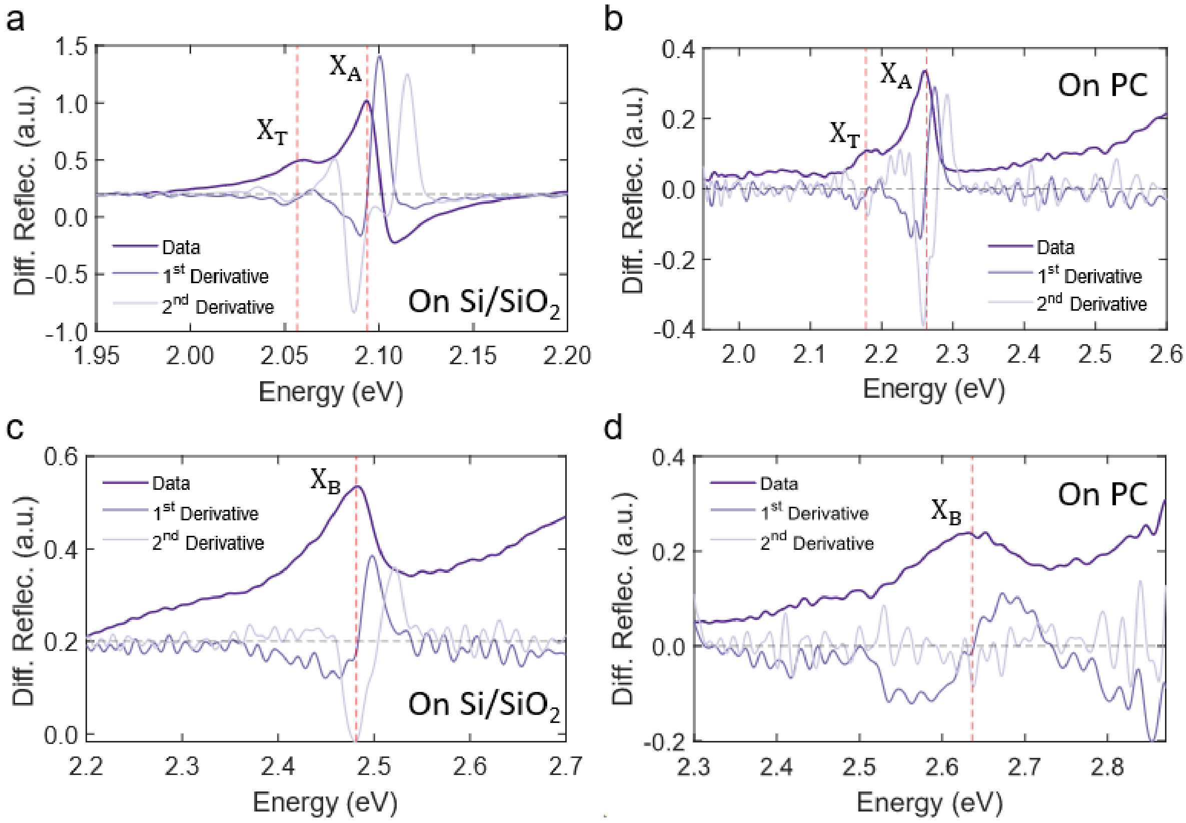

Figure S5. Determination of the energy position of exciton resonances at 10 K for a single layer WS_2 sample using the criteria of the first and second derivative. a-b) The red dashed lines indicate the position of the resonance corresponding to X_T and X_A excitons for 1L-WS_2 deposited on Si/SiO_2 (top-left) and polycarbonate (top-right) substrates. c-d) The red dashed lines indicate the position of the peak corresponding to X_B exciton for 1L-WS_2 deposited on Si/SiO_2 (bottom-left) and polycarbonate (bottom-right) substrates.

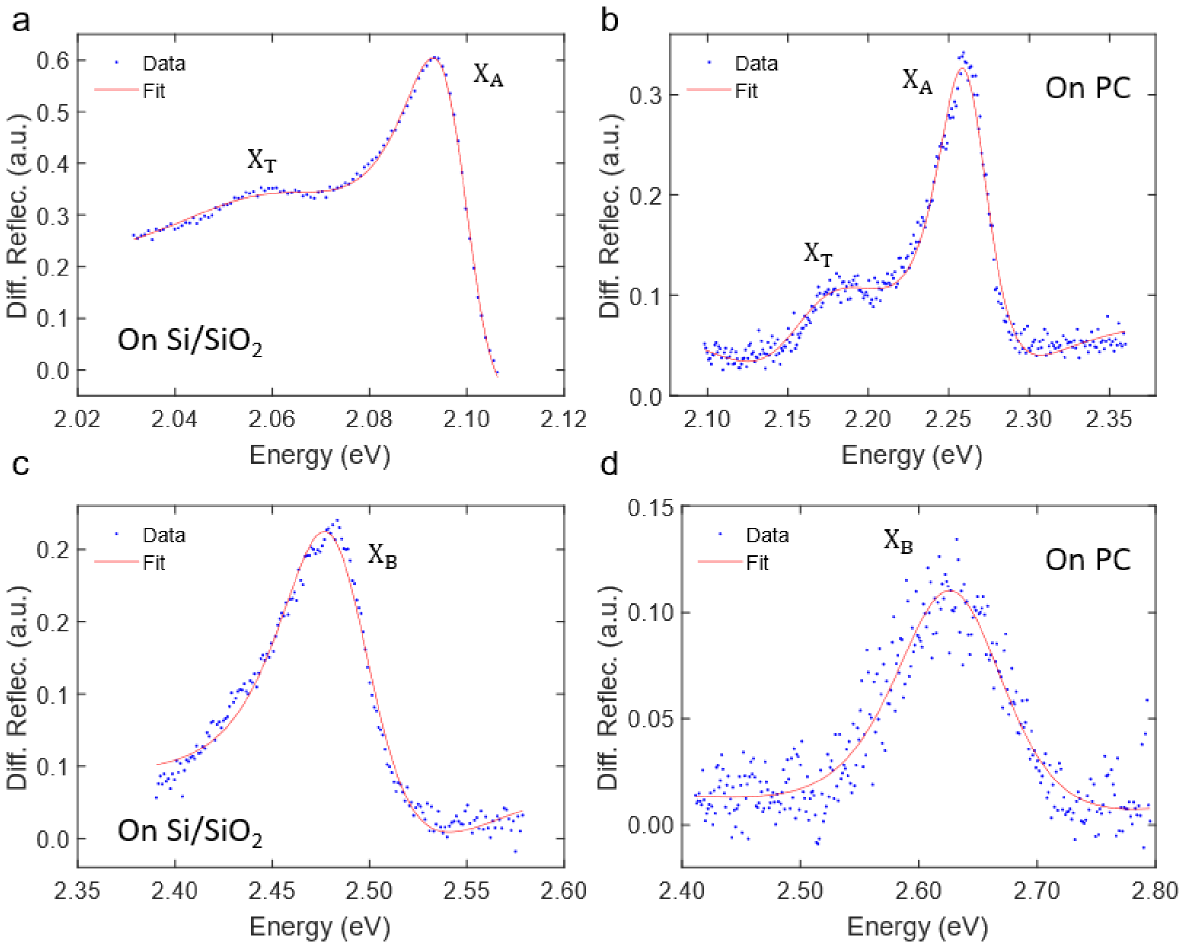

Figure S6. Determination of the energy position of exciton peaks at 10 K for 1L-WS_2 samples by fitting the differential reflectance spectra to the Aspnes formula (eq. S1). a-b) Data is fitted to the sum of two resonances corresponding to X_T and X_A excitons for (a) 1L-WS_2 deposited on Si/SiO_2 and (b) polycarbonate substrates. c-d) Data is fitted to one single resonance corresponding to X_B exciton for 1L-WS_2 deposited on (c) Si/SiO_2 and (d) polycarbonate substrates.

Following Ref. [s4], the moduli for resonances X_A and X_T are given by the expression

$$\xi(E) = \frac{|C_j|}{(E-E_j)^2+\Gamma_j^2} \qquad \text{(eq. S2)}$$

which is a Lorentzian line shape with the same parameters as those extracted from the fit to the Aspnes equation.

The relative spectral weight between exciton and trion can be directly related to the doping level of 1L-TMDs (see for example Ref. [s5]). From our data, the relative spectral weight between exciton and trion can be estimated as the ratio of the areas under the resonance moduli described by eq. S2. Results for the relative weight of trion versus excitons for 1L-WS_2 and 1L-MoS_2 are presented in Table S3. The increase of trion relative

weight suggests an increase of doping level for samples on PC compared to those on Si/SiO_2.

Table S2. Fit parameters for 1L-WS_2 and 1L-MoS_2 differential reflectance data in the energy range of resonances X_A and X_T to the Aspnes expression (eq. S1). The area under the resonance moduli (eq. S2) is calculated from (eq. S2) as $Area = \frac{\pi C}{\Gamma}$.

**1L-WS_2 on Si/SiO_2**	**C (a.u)**	**θ**	**E (eV)**	**Γ (m eV)**	**Area (a.u.)**
X_A	4.99E-5	0.46	2.10	10.6	1.48E-5
X_T	9.22E-6	0.88	2.06	14.7	1.97E-6
**1L-WS_2 on PC**	**C (a.u)**	**θ**	**E (eV)**	**Γ (meV)**	**Area (a.u.)**
X_A	1.73E-4	0.65	2.27	27.1	2.00E-5
X_T	3.54E-5	0.94	2.18	31.4	3.54E-6
**1L-MoS_2 on Si/SiO_2**	**C (a.u)**	**θ**	**E (eV)**	**Γ (meV)**	**Area (a.u.)**
X_A	2.36E-5	0.70	1.96	14.8	5.01E-6
X_T	1.00E-5	0.93	1.93	16.3	1.93E-6
**1L-MoS_2 on PC**	**C (a.u)**	**θ**	**E (eV)**	**Γ (meV)**	**Area (a.u.)**
X_A	4.05E-5	0.82	2.10	26.0	4.89E-6
X_T	6.18E-5	0.90	2.06	25.5	7.61E-6

Table S3. Ratio between the areas (A_X) under X_A and X_T resonances (table S2) for 1L-WS_2 and 1L-MoS_2 deposited on Si/SiO_2 and PC substrates, respectively.

**1L-WS_2 on Si/SiO_2**	**A_{XT} / A_{XA}**<br>0.13	**1L-MoS_2 on Si/SiO_2**	**A_{XT} / A_{XA}**<br>0.39
**1L-WS_2 on PC**	**A_{XT} / A_{XA}**<br>0.18	**1L-MoS_2 on PC**	**A_{XT} / A_{XA}**<br>1.56

S4. Parameters from fitting exciton energies in 1L-TMDs on Si/SiO_2 to the O'Donell model for bandgap evolution with temperature

O'Donell *et al.*, Ref. [s6], proposed a phenomenological model to describe the evolution of the bandgap energy of a semiconductor with temperature:

$$E_g(T) = E_g(0) - S\langle\hbar\omega\rangle[\coth(\langle\hbar\omega\rangle/2kT) - 1] \quad \text{(Eq. S3)}$$

where $E_g(0)$ is the band gap energy at zero temperature, S is a dimensionless coupling constant and $\langle\hbar\omega\rangle$ is the averaged phonon energy. Table S4 displays the fitting parameters obtained from fitting the exciton energies as a function of temperature (Figure 3 in the main text) to equation S3. The resulting fits are plotted with a solid line in the Figure 3 of the main text.

Table S4. Fitting parameters of exciton energy positions as a function of temperature to the O'Donell expression (Eq. s3). Error is estimated from 95% confidence bounds

**1L-WS_2**	X_A	X_B	X_T
E_0 (eV)	2.091 ± 0,001	2.479 ± 0.002	2.054 ± 0.001
S	2.4 ± 0,1	2.5 ± 0.1	2.3 ± 0.3
h**ω** (meV)	22 ± 1	18 ± 3	19 ± 4

**1L-MoS_2**	X_A	X_B	X_T
E_0 (eV)	1.958 ± 0.002	2.086 ± 0.004	1.931 ± 0.002
S	2.3 ± 0.1	2.3 ± 0.3	1.5 ± 0.2
h**ω** (meV)	28 ± 4	31 ± 5	8 ± 5

**1L-WSe_2**	X_A	X_B
E_0 (eV)	1.742 ± 0.001	2.164 ± 0.003
S	2.7 ± 0.1	2.6 ± 0.3
h**ω** (meV)	21 ± 2	16 ± 5

**1L-$MoSe_2$**	X_A	X_B
E_0 (eV)	1.656 ± 0.001	1.862 ± 0.003
S	2.8 ± 0.2	2.4 ± 0.2
h**ω** (meV)	24 ± 3	18 ± 4

S5. E_X^{PC} - $E_X^{Si/SiO2}$ as a function of temperature

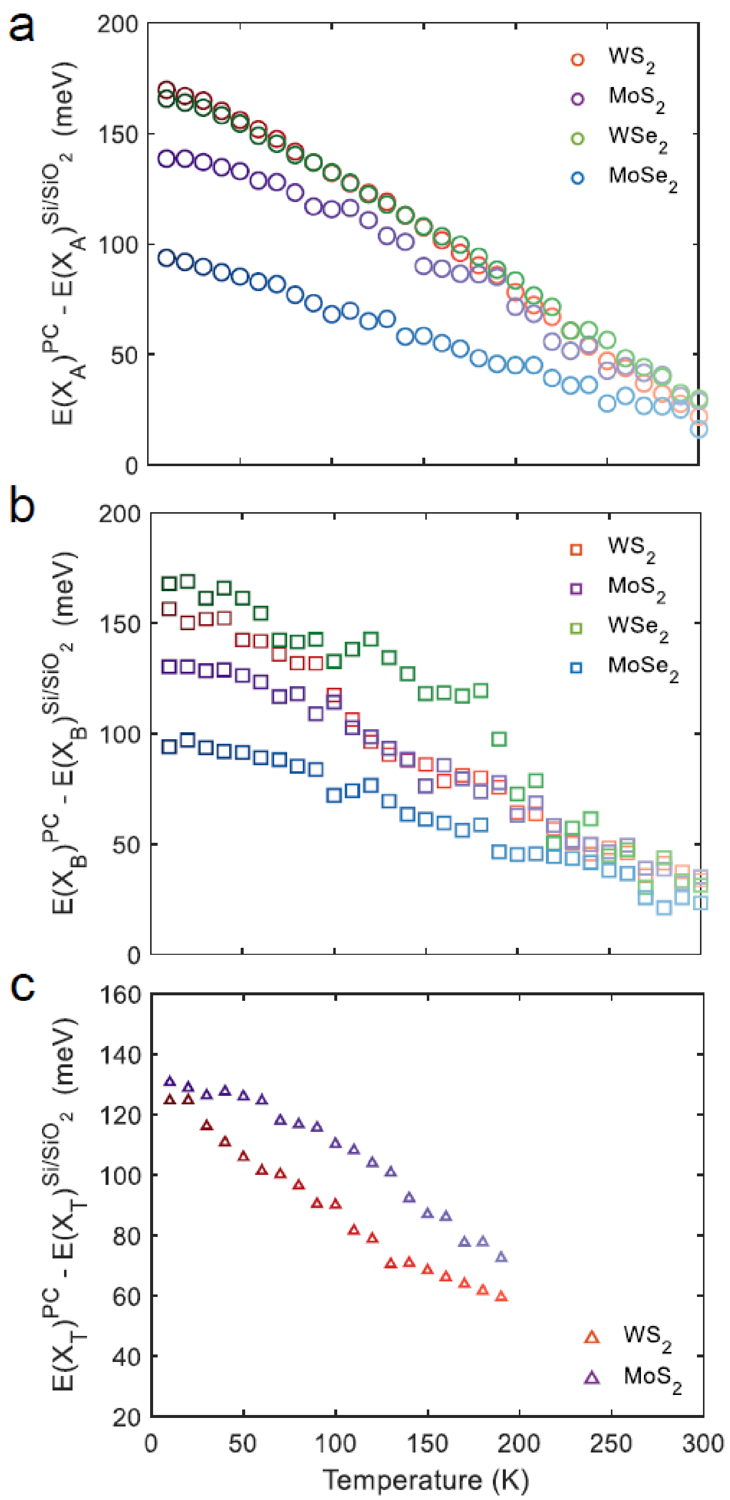

Figure S7. a-c) Markers represent the difference ΔE(X) = E_X^{PC} - $E_X^{Si/SiO2}$ between the energies of (a) X_A excitons, (b) X_B excitons and (c) X_T charged excitons on the two different substrates, PC and Si/SiO_2, as a function of temperature, for a single layer of each of the four materials under study.

S6. Linear fitting analysis of $E(X)^{PC}$- $E(X)^{Si/SiO2}$ with substrate deformation for X_A, X_B and X_T

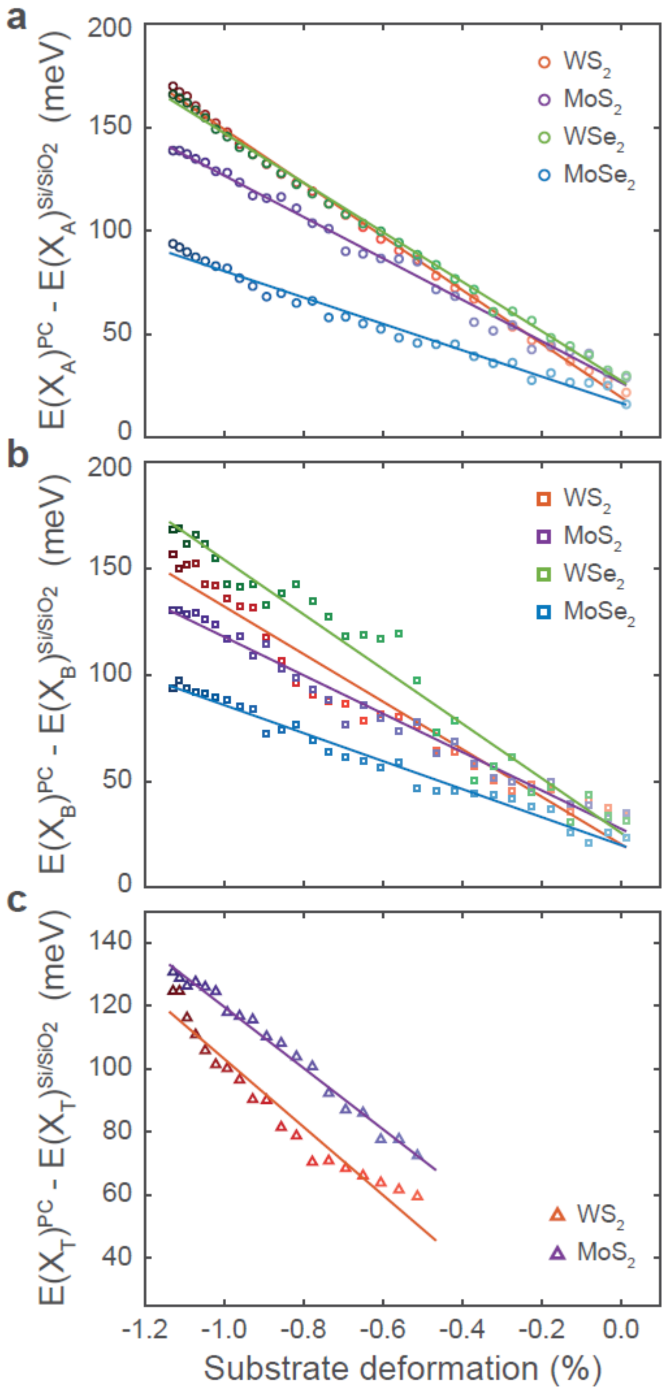

Figure S8. Markers represent the energy difference $\Delta E(X) = E_X^{PC} - E_X^{Si/SiO2}$ between a) X_A, b) X_B and c) X_T excitons on the two different substrates, PC and Si/SiO$_2$, as a function of substrate deformation (estimated as $\Delta L/L$ in Fig. 1c in the main text), for a single layer of each of the four materials under study. Solid lines represent linear fits to the data summarized in Table S5. The slope of these linear fits yields the gauge factors presented in Table 1 in the main text. The *y*-intercept value represents the exciton energy difference between substrates at room temperature, that is, in the absence of strain.

Table S5. Results from the linear fit of exciton energy data with substrate deformation (see Figure S8): ΔE = GF*x + ΔE_0, where x represents substrate deformation. The slope is the strain gauge factor. ΔE_0 represents the energy difference between substrates at room temperature in the absence of strain. Error is estimated from 95% confidence bounds.

**1L TMD**	**X_A**		**X_B**		**X_T**	
	Gauge factor (meV/%)	ΔE_0 (meV)	Gauge factor (meV/%)	ΔE_0 (meV)	Gauge factor (meV/%)	ΔE_0 (meV)
WS_2	-129 ± 2	20 ± 2	-110 ± 10	20 ± 7	-110 ± 13	5 ± 2
MoS_2	-100 ± 4	27 ± 3	-90 ± 5	28 ± 4	-97 ± 5	23 ± 5
WSe_2	-120 ± 2	28 ± 2	-130 ± 10	26 ± 7	--	--
$MoSe_2$	-64 ± 3	17 ± 3	-66 ± 4	20 ± 3	--	--

S7. Gauge factors for additional 1L-TMDs samples

Table S6. Summary of strain gauge factors obtained for various single-layer TMD samples under different cooldown and warm-up cycles from 300 to 10 K. Gauge factors are obtained from the linear fit of $\Delta E(X) = E_X^{PC} - E_X^{S/SiO2}$ against substrate deformation, as detailed in the main text. Error for each value is estimated from 95% confidence bounds. The first row for each material corresponds to the set of data presented and analyzed in the main text. The mean and standard deviation values for all measurements performed for each material are also presented.

**1L TMD**	Cycle	X_A	X_B	X_T
		**Gauge Factor** (meV/%)	**Gauge Factor** (meV/%)	**Gauge Factor** (meV/%)
Samp. 1 **WS_2**	Cooldown	-129 ± 2	-112 ± 10	-108 ± 13
	Warmup	-130 ± 2	-127 ± 17	-102 ± 13
Samp. 2 **WS_2**	Cooldown	-128 ± 2	-117 ± 15	-94 ± 14
	Warmup	-129 ± 2	-110 ± 9	-100 ± 12
Samp. 3 **WS_2**	Cooldown	-141 ± 5	-109 ± 13	-113 ± 16
	Warmup	-141 ± 3	-113 ± 11	-123 ± 17
Samp. 4 **WS_2**	Cooldown	-125 ± 6	-101 ± 15	-105 ± 10
**Mean value ± STD**		**-132 ± 7**	**-113 ± 6**	**-108 ± 10**
Samp. 1 **MoS_2**	Cooldown	-100 ± 4	-90 ± 5	-97 ± 5
	Warmup	-100 ± 5	-91 ± 6	-88 ± 9
Samp. 2 **MoS_2**	Cooldown	-88 ± 6	-65 ± 5	-85 ± 4
**Mean value ± STD**		**-96 ± 7**	**-82 ± 15**	**-90 ± 7**
Samp. 1 **WSe_2**	Cooldown	-120 ± 2	-129 ± 10	--
	Warmup	-117 ± 2	-127 ± 6	--
Samp. 2 **WSe_2**	Cooldown	-118 ± 4	-121 ± 10	--
	Warmup	-115 ± 4	-127 ± 10	--
**Mean value ± STD**		**-117 ± 2**	**-126 ± 4**	**--**
Samp. 1 **$MoSe_2$**	Cooldown	-64 ± 3	-66 ± 4	--
	Warmup	-64 ± 4	-64 ± 4	--
Samp. 2 **$MoSe_2$**	Cooldown	-67 ± 3	-68 ± 3	--
**Mean value ± STD**		**-65 ± 2**	**-67 ± 2**	**--**

S8. A comparative of strain gauge factors for excitons in 1L-TMDs

A. Gauge factors for tensile and compressive strain induced by thermal expansion or compression or the use of piezoelectric substrates

Table S7. Gauge factors for Exciton *A* in 1L-TMDs under tensile and compressive strain induced by substrate thermal expansion/compression.

Reference	Substrate/ Temperature	Uniaxial/ biaxial Tensile/ compressive	Max. Strain range (%)	Gauge factor 1L-WS_2 (meV/%)	Gauge factor 1L-MoS_2 (meV/%)	Gauge factor 1L-WSe_2 (meV/%)	Gauge factor 1L-$MoSe_2$ (meV/%)
**This work**	**Polycarbonate (10-300K)**	**Biaxial compressive**	**-1.2 to 0**	**129**	**100**	**120**	**64**
Plechinger *et al*. 2D Mater. 2015 [s7]	PDMS (300-400K)	Biaxial tensile	0 to 0.2	–	105*	–	–
Frisenda *et* al. npj 2D Mater Appl 2017 [s8]	Polypropylene (300-375K)	Biaxial tensile and compressive	-0.1 to 1	94* (~60)	51.1* (~30)	63* (~40)	33* (~20)
Gant *et al*. Materials Today 2019 [s9]	Polycarbonate (75-375K)	Biaxial tensile and compressive	-1.5 to 0.5	–	135* (~65)	–	–
Ryu *et al*. Nano Lett. 2020, [s10]	Polypropylene (300-350K)	Biaxial tensile	0 to 0.64	–	74* (48)	–	–

*Gauge factor value without subtraction of temperature effects on the bandgap energy shifts. Within brackets are the estimated values once the temperature effects are removed, using the same method applied to our data and described in the main text.

Table S8. Gauge factors for Exciton *A* in 1L-TMDs under strain induced by piezo-electric substrate expansion/compression.

Reference	Substrate/ Temperature	Uniaxial/ biaxial Tensile/ compressive	Max. Strain range (%)	Gauge factor 1L-WS_2 (meV/%)	Gauge factor 1L-MoS_2 (meV/%)	Gauge factor 1L-WSe_2 (meV/%)	Gauge factor 1L-$MoSe_2$ (meV/%)
Iff et al. Nano Lett. 2019 [s19]	Piezoelectric actuator	Uniform tensile and **compressive**	-0.15 to 0.15			33	
An et al. PRB 2023 [s20]	Piezoelectric actuator	Uniform tensile and **compressive**	-0.1 to 0.1			80	50

B. Gauge factors for tensile biaxial strain induced by other methods

Table S9. Comparative of Gauge factors for Exciton *A* under biaxial tensile strain induced by different methods.

Reference	Method	Tensile/ compressive	Max. Strain range (%)	1L-WS2 (meV/%)	1L-MoS2 (meV/%)	1L-WSe2 (meV/%)	1L-MoSe2 (meV/%)
Michail, *et al.* Phys. Chem. C, 2023 [s11]	Bending on PMMA cruciform	Tensile	0 to 0.7	130	–	–	–
Michail *et al.*, 2D Materials, 2021 [s12]	Bending on PMMA cruciform	Tensile	0 to 0.6	–	Exfoliated 124 ± 17 CVD growth 76 ± 10	–	–
Carrascoso *et al.,* Nano Mat. Science, 2022 [s13]	Bending on Mylar cruciform	Tensile	0 to 1.04	–	108	–	–
Lloyd *et al.*, Nano Letters, 2016 [s14]	Pressurized membranes	Non-uniform Tensile	0 to 5.6	--	99	--	--
Covre, F.S., Nanoscale,2022 [s15]	Bubbles	Non-uniform Tensile	0 to 1.3	--	--	--	54
Harats, M.G., Nat. Photonics, 2020 [s16]	Nano-indentation	Non-uniform Tensile	0 to 2.8	30-40	--	--	--
Blundo, E., Phys. Rev. Res., 2020 [s17]	H_2-filled Domes	Non-uniform Tensile	0 to 2.1	45	37	--	--
Chen, Z., ACS Applied Nano Mat., 2022 [s18]	Micro-pillars	Non-uniform Tensile	0 to 0.7	--	--	50	--

S9. Differential reflectance for 1L-WS_2 on a Polypropylene substrate

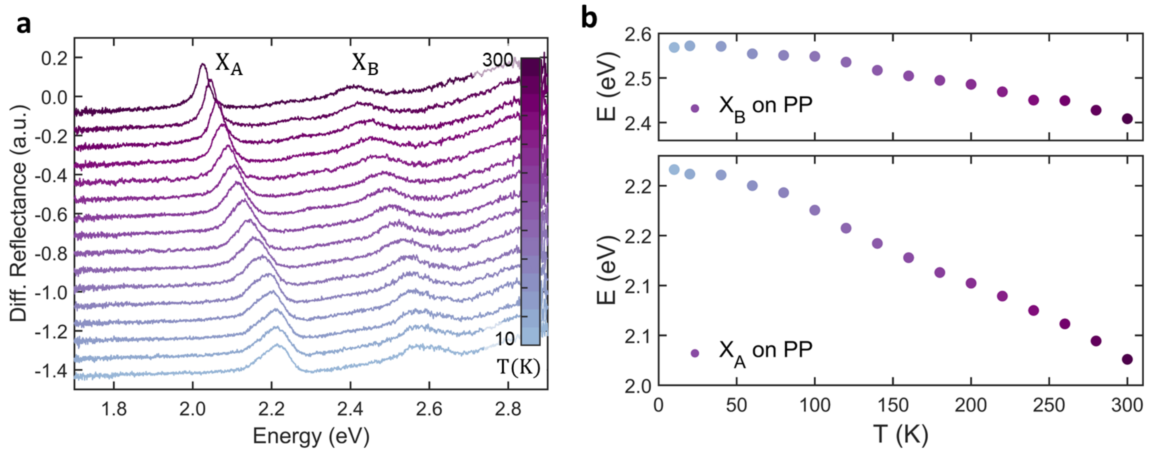

Figure S9. a) Differential reflectance as a function of temperature for 1L-WS_2 on a Polypropylene (PP) substrate. (b) Markers represent the energy positions for resonances X_B (top) and X_A (bottom) extracted from (a).

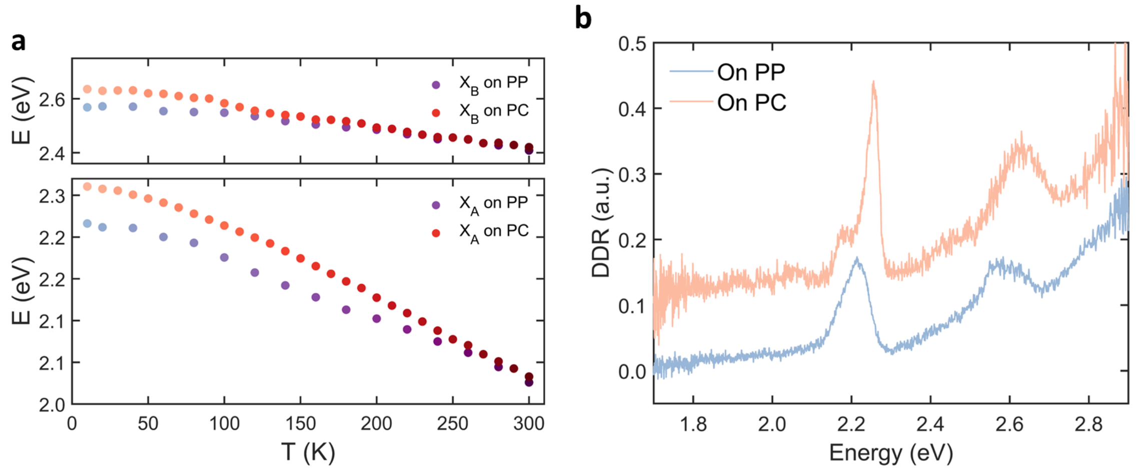

Figure S10. Comparison of the measurements performed on PP with the measurements performed on PC. (a) Markers represent the energy positions for resonances X_B (top) and X_A (bottom) extracted from differential reflectance as a function of temperature for 1L-WS_2 deposited on Polypropylene (PP) and on Polycarbonate (PC), respectively. (b) Differential reflectance at 10 K for single layer WS_2 samples on PP (red line) and on PC (blue line).

Table S10 displays the temperature gauge factors obtained for *A* and *B* excitons for single-layer WS_2 samples deposited on PP and on PC, respectively, at low temperature. Despite polypropylene having a higher expansion coefficient compared to polycarbonate (see Table S11), the gauge factor obtained for excitons *A* and *B* in 1L-WS_2 deposited on PC is slightly smaller than when it is deposited on PP.

Table S10. Temperature Gauge factors for the energy positions of excitonic resonances

Gauge factor (meV/K)	X_A	X_B
PP	188 ± 4	161 ± 12
PC	226 ± 3	212 ± 10

Table S11. Comparison of thermal, mechanical and dielectrics properties for different polymers.

**Polymer**	**Thermal expansion coefficient at RT (K^{-1})**	**Thermal conductivity (W/mK)**	**Young's modulus (GPa)**	**Dielectric Constant**
Polycarbonate (PC)	6,5 x10^{-5} [s9]<br>6–17 x10^{-5} [s25]	0.20 [s25]	~ 2.5 [s24]<br>2,1-2,5 [s25]	2,8 [s27]<br>3,0 [s25]
Polypropylene (PP)	~13 x10^{-5} [s24]<br>6–17 x10^{-5} [s25]	0.15-0.21 [s25]	1.5–2,0 [s24]<br>1,1-1,6 [s25]	2,3 [s25]
Polyethylene terephthalate (PET)	6–8 x10^{-5} [s25]	0.29 [s25]	2,016 [s26]<br>2,8-3,5 [s25]	3-4 [s25]

S10. Trion binding energies as a function of temperature

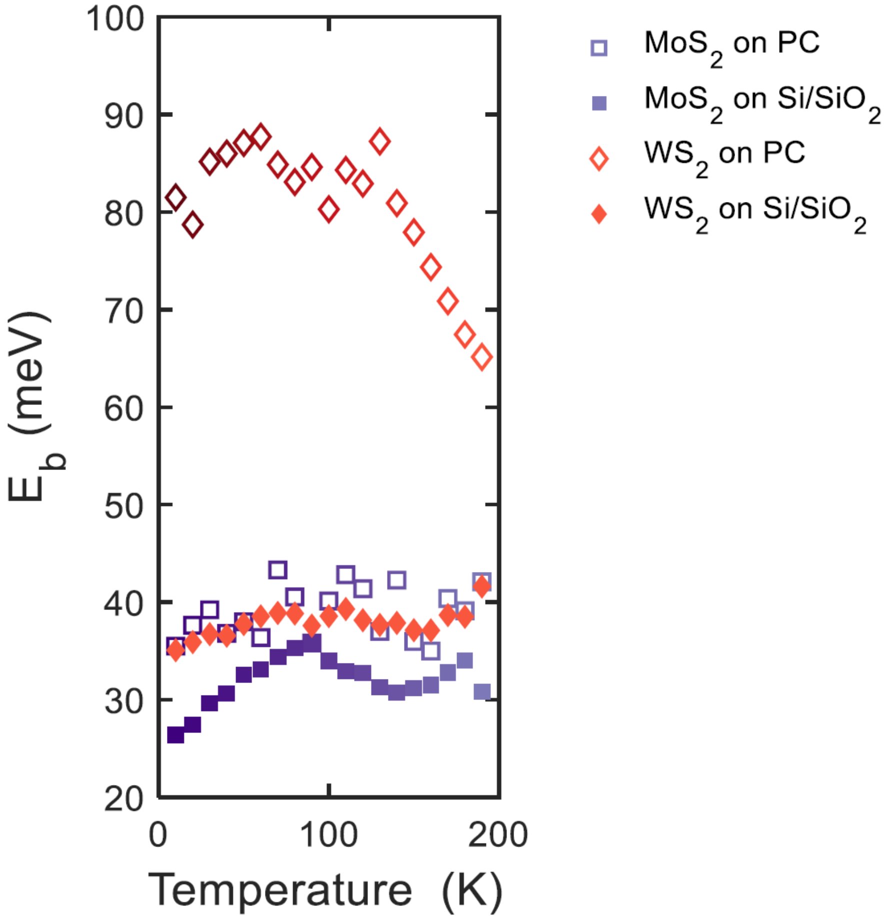

Figure S11. Energy difference E_b = $E(X_A)$ - $E(X_T)$ between X_T and X_A in the temperature range where X_T can be resolved for 1L-MoS_2 (square) and 1L-WS_2 (diamond) both on PC and on Si/SiO_2 (filled). This energy difference is associated to the binding energy of a charged exciton.

S11. Exciton reduced mass as a function of strain from DFT calculations

The exciton reduced mass is defined as $\mu = \frac{m_e^* m_h^*}{m_e^* + m_h^*}$, where m_e^* and m_h^* are the electron and hole effective masses, respectively. Effective masses are obtained from DFT calculations, as described in the Methods sections of the manuscript.

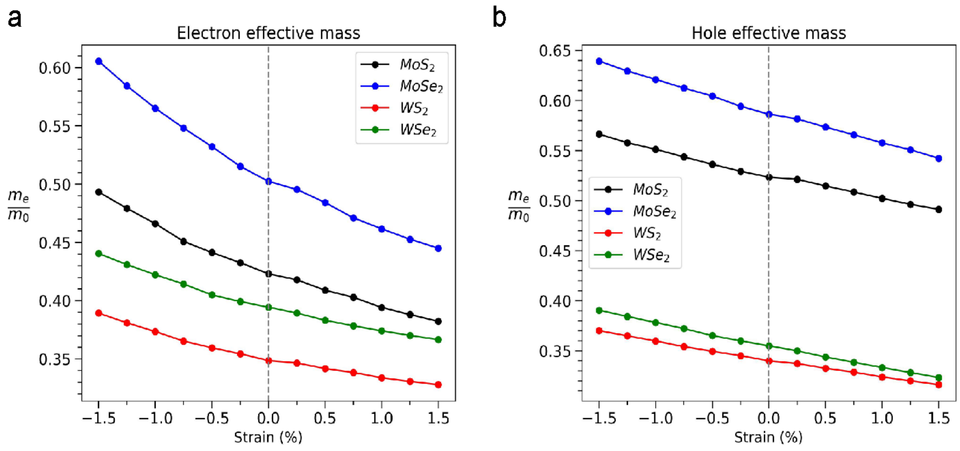

Figure S12. Evolution of effective masses with strain for the electrons in the first conduction band (panel a) and the holes of the last valence band (panel b) for the TMDC studied in the main text.

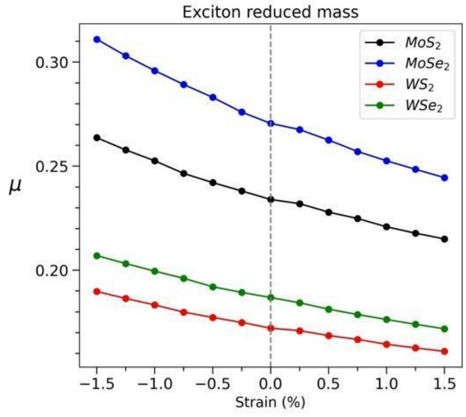

Figure S13. Evolution of the excitons reduced mass with strain calculated from the effective masses presented in Figure S10.

S12. Trion binding energy estimations

Negative trion binding energies are estimated for a 1.5% degree of compressive biaxial strain. Two different methods are employed to estimate the binding energies: a) from the code provided by Szyniszewski et al. [s21], which implements a diffusion quantum MonteCarlo method and allows for the evaluation of exciton and trion binding energies accounting for the dielectric influence of the substrate; b) only in vacuum, from the values reported in Berkelbach et al. [s22] using a variational method. In both cases, the trion binding energy depends on effective masses for electrons and holes and on the screening lengths for the attractive potential within the material. Input values for effective masses are extracted from the ab-initio calculations described in section S11. Screening lengths are obtained from two different sources: a) by extrapolating the polarizability values provided in Ref. [s23] in Tables S12 and S14 for 1L-MoS_2 and 1L-WS_2, respectively; (b) by assuming a similar variation with strain of the polarizability values provided in Ref. [s22] in Tables S13 and S15 for 1L-MoS_2 and 1L-WS_2, respectively. The relative dielectric constant ε_r is estimated as the average value of the chosen substrate and vacuum, with $\varepsilon_r(SiO_2) = 3.9$ and $\varepsilon_r(PC)=2.8$. The relation between screening length ρ and polarizability χ_{2D} is given by $\rho=2\pi\chi_{2D}$ or by $\rho=2\pi\chi_{2D}/\varepsilon_r$ in the case of considering the dielectric screening from substrates [s21].

Since energies vary substantially depending on the chosen approximation, in order to provide an upper bound for the change of negative trion binding energies due to strain, we also consider an overestimation of the effects of strain. This involves assuming a 20% increase in the reduced mass and a 10% decrease in the polarizability.

Table S12. Effective mass from ab-initio calculations (section S11) and screening length parameters (from Ref. [s23]) for **1L-MoS_2** under compressive biaxial strain. Trion binding energies are extracted using the code provided in Ref. [s21] and from data published in Ref. [s22]. The extreme values representing the maximum difference in trion energies, considering both strain and the dielectric screening from different substrates, are marked in bold.

**Biaxial Strain (%)**	**m^*_e/m_0**	**m^*_h/m_0**	**Reduced mass (μ)**	**Polarizability χ_{2D}** (Å) [s23]	**Trion E_b vacuum (meV)** [s22]	**Trion E_b vacuum (meV)** (ε_r = 1) [s21]	**Trion E_b SiO_2 (meV)** (ε_r = 2.45) [s21]	**Trion E_b PC (meV)** (ε_r= 1.9) [s21]
0	0.423	0.523	0.234	6.989	24.3	32.6	**22.0**	25.0
-1.5	0.493	0.566	0.264 (+12%)	6.75* (-4%)	25.6	34.3	23.3	26.4
Over-estimation of strain effects	(+20%) 0.508	(+20%) 0.628	(+20%) 0.281	(-10%) 6.3	27.5	36.7	24.8	**28.3**

*Extrapolated from the values provided in Ref. [s23]

Table S13. Effective mass from ab-initio calculations (section S11) and screening length parameters (from Ref. [s22]) for **1L-MoS_2** under compressive biaxial strain. Trion binding energies are extracted using the code provided in Ref. [s21] and from data published in Ref. [s22]. The extreme values representing the maximum difference in trion energies, considering both strain and the dielectric screening from different substrates, are marked in bold.

**Biaxial Strain (%)**	**m^*_e/m_0**	**m^*_h/m_0**	**Reduced mass (μ)**	**Polarizability χ_{2D}** (Å) [s22]	**Trion E_b vacuum (meV)** [s22]	**Trion E_b vacuum (meV)** (ε_r = 1) [s21]	**Trion E_b SiO_2 (meV)** (ε_r = 2.45) [s21]	**Trion E_b PC (meV)** (ε_r= 1.9) [s21]
0	0.423	0.523	0.234	6.6	25.4	34.6	**23.3**	26.5
-1.5	0.493	0.566	0.264 (+12%)	6.27 (-5%)	27.4	36.9	24.9	28.3
Over-estimation of strain effects	(+20%) 0.508	(+20%) 0.628	(+20%) 0.281	5.94 (-10%)	29.0	39	26.4	**29.9**

Table S14. Effective mass from ab-initio calculations (section S11) and screening length parameters (from Ref. [s23]) for **1L-WS_2** under compressive biaxial strain. Trion binding energies are extracted using the code provided in Ref. [s21] and from data published in Ref. [s22]. The extreme values representing the maximum difference in trion energies, considering both strain and the dielectric screening from different substrates, are marked in bold.

**Biaxial Strain (%)**	**m^*_e/m_0**	**m^*_h/m_0**	**Reduced mass (μ)**	**Polarizability χ_{2D}** (Å) [s23]	**Trion E_b vacuum (meV)** [s22]	**Trion E_b vacuum (meV)** (ε_r = 1) [s21]	**Trion E_b SiO_2 (meV)** (ε_r = 2.45) [s21]	**Trion E_b PC (meV)** (ε_r= 1.9) [s21]
0	0.348	0.340	0.172	6.462	24.5	32.7	**21.4**	24.6
-1.5	0.389	0.370	(+10%) 0.190	6.371* (-2%)	25.6	33.8	22.2	25.5
Over-estimation of strain effects	(+20%) 0.416	(+20%) 0.408	(+20%) 0.206	(-10%) 5.81	28.0	36.9	24.3	**27.9**

*extrapolated from the values provided in Ref. [s23]

Table S15 Effective mass from ab-initio calculations (section S11) and screening length parameters (from Ref. [s20]) for **1L-WS_2** under compressive biaxial strain. Trion binding energies are extracted using the code provided in Ref. [s19] and from data published in Ref. [s20]. The extreme values representing the maximum difference in trion energies, considering both strain and the dielectric screening from different substrates, are marked in bold.

**Biaxial Strain (%)**	**m^*_e/m_0**	**m^*_h/m_0**	**Reduced mass (μ)**	**Polarizability χ_{2D}** (Å) [s22]	**Trion E_b vacuum (meV)** [s22]	**Trion E_b vacuum (meV)** (ε_r = 1) [s21]	**Trion E_b SiO_2 (meV)** (ε_r = 2.45) [s21]	**Trion E_b PC (meV)** (ε_r= 1.9) [s21]
0	0.348	0.340	0.172	6.0	26.2	34.8	**22,5**	26
-1.5	0,39	0,37	(+10%) 0.190	(-5%) 5.7	27.9	36.9	24	27,7
Over-estimation of strain effects	(+20%) 0,42	(+20%) 0.408	(+20%) 0.207	(-10%) 5.4	29.4	39,2	25,6	**29,5**

Supporting references

[s1] Niu, Y., Gonzalez-Abad, S., Frisenda, R., Marauhn, P., Drüppel, M., Gant, P., ... & Castellanos-Gomez, A. Thickness-dependent differential reflectance spectra of monolayer and few-layer MoS_2, $MoSe_2$, WS_2 and WSe_2. *Nanomaterials*, 2018, 8(9), 725.

[s2] Yuan, L., & Huang, L. Exciton dynamics and annihilation in WS_2 2D semiconductors. *Nanoscale*, 2015, 7(16), 7402-7408.

[s3] Aspnes, D. E. . Third-derivative modulation spectroscopy with low-field electroreflectance. *Surface science,* 1973, 37, 418-442. 1973

[s4] Kopaczek, J., Zelewski, S., Yumigeta, K., Sailus, R., Tongay, S., & Kudrawiec, R. Temperature dependence of the indirect gap and the direct optical transitions at the high-symmetry point of the brillouin zone and band nesting in MoS_2, $MoSe_2$, $MoTe_2$, WS_2, and WSe_2 crystals. *The Journal of Physical Chemistry C*, 2022, 126(12), 5665-5674.

[s5] Mouri, S.; Miyauchi, Y.; Matsuda, K. Tunable Photoluminescence of Monolayer MoS_2 via Chemical Doping. *Nano Lett.,* 2013, 13 (12), 5944–5948.

[s6] O'Donnell, K. P.; Chen, X. Temperature Dependence of Semiconductor Band Gaps. *Appl. Phys. Lett.,* 1991, *58* (25), 2924–2926.

[s7] Plechinger, G.; Castellanos-Gomez, A.; Buscema, M.; Zant, H. S. J. van der; Steele, G. A.; Kuc, A.; Heine, T.; Schüller, C.; Korn, T. Control of Biaxial Strain in Single-Layer Molybdenite Using Local Thermal Expansion of the Substrate. *2D Mater.,* 2015, 2 (1), 015006.

[s8] Frisenda, R.; Drüppel, M.; Schmidt, R.; Michaelis de Vasconcellos, S.; Perez de Lara, D.; Bratschitsch, R.; Rohlfing, M.; Castellanos-Gomez, A. Biaxial Strain Tuning of the Optical Properties of Single-Layer Transition Metal Dichalcogenides. *npj 2D Mater Appl,* 2017, *1* (1), 10

[s9] Gant, P.; Huang, P.; Pérez de Lara, D.; Guo, D.; Frisenda, R.; Castellanos-Gomez, A. A Strain Tunable Single-Layer MoS2 Photodetector. *Materials Today,* 2019, *27*, 8–13.

[s10] Ryu, Y. K.; Carrascoso, F.; López-Nebreda, R.; Agraït, N.; Frisenda, R.; Castellanos-Gomez, A. Microheater Actuators as a Versatile Platform for Strain Engineering in 2D Materials. *Nano Lett.,* 2020, *20* (7), 5339–5345

[s11] Michail, A., Anestopoulos, D., Delikoukos, N., Grammatikopoulos, S., Tsirkas, S. A., Lathiotakis, N. N., ... & Papagelis, K. Tuning the Photoluminescence and Raman Response of Single-Layer WS_2 Crystals Using Biaxial Strain. *The Journal of Physical Chemistry C*, 2023, 127(7), 3506-3515.

[s12] Michail, A., Anestopoulos, D., Delikoukos, N., Parthenios, J., Grammatikopoulos, S., Tsirkas, S. A., ... & Papagelis, K..Biaxial strain engineering of CVD and exfoliated single-and bi-layer MoS_2 crystals. *2D Materials*, 2020, 8(1), 015023.

[s13] Carrascoso, F., Frisenda, R., & Castellanos-Gomez, A. Biaxial versus uniaxial strain tuning of single-layer MoS_2. *Nano Materials Science*, 2022, 4(1), 44-51.

[s14] Lloyd, D., Liu, X., Christopher, J. W., Cantley, L., Wadehra, A., Kim, B. L., ... & Bunch, J. S. Band gap engineering with ultralarge biaxial strains in suspended monolayer MoS_2. *Nano Letters*, 2016, 16(9), 5836-5841.

[s15] Covre, F. S., Faria, P. E., Gordo, V. O., de Brito, C. S., Zhumagulov, Y. V., Teodoro, M. D., ... & Gobato, Y. G. Revealing the impact of strain in the optical properties of bubbles in monolayer $MoSe_2$. *Nanoscale*, 2022, 14(15), 5758-5768.

[s16] Harats, M. G.; Kirchhof, J. N.; Qiao, M.; Greben, K.; Bolotin, K. I. Dynamics and Efficient Conversion of Excitons to Trions in Non-Uniformly Strained Monolayer WS2. *Nat. Photonics,* 2020, *14* (5), 324–329.

[s17] Blundo, E., Felici, M., Yildirim, T., Pettinari, G., Tedeschi, D., Miriametro, A., ... & Polimeni, A. Evidence of the direct-to-indirect band gap transition in strained two-dimensional WS_2, MoS_2, and WSe_2. *Physical Review Research*, 2020, 2(1), 012024.

[s18] Chen, Z., Luo, W., Liang, L., Ling, X., & Swan, A. K. Charge Separation in Monolayer WSe2 by Strain Engineering: Implications for Strain-Induced Diode Action. *ACS Applied Nano Materials*, 2022, 5(10), 15095-15101.

[s19] Iff, O.; Tedeschi, D.; Martín-Sánchez, J.; Moczała-Dusanowska, M.; Tongay, S.; Yumigeta, K.; Taboada-Gutiérrez, J.; Savaresi, M.; Rastelli, A.; Alonso-González, P.; Höfling, S.; Trotta, R.; Schneider, C. Strain-Tunable Single Photon Sources in WSe2 Monolayers. *Nano Lett.,* 2019, *19* (10), 6931–6936.

[s20] An, Z.; Soubelet, P.; Zhumagulov, Y.; Zopf, M.; Delhomme, A.; Qian, C.; Junior, P. E. F.; Fabian, J.; Cao, X.; Yang, J.; Stier, A. V.; Ding, F.; Finley, J. J. Strain Control of Exciton and Trion Spin-Valley Dynamics in Monolayer Transition Metal Dichalcogenides. *Physical Review B* 2023, 108, L041404.

[s21] Szyniszewski, M.; Mostaani, E.; Drummond, N. D.; Fal'ko, V. I. Binding Energies of Trions and Biexcitons in Two-Dimensional Semiconductors from Diffusion Quantum Monte Carlo Calculations. *Phys. Rev. B,* 2017, *95* (8), 081301.

[s22] Berkelbach, T. C.; Hybertsen, M. S.; Reichman, D. R. Theory of Neutral and Charged Excitons in Monolayer Transition Metal Dichalcogenides. *Phys. Rev. B,* 2013, *88* (4), 045318.

[s23] Wang, L.; Kutana, A.; Yakobson, B. I. Many-Body and Spin-Orbit Effects on Direct-Indirect Band Gap Transition of Strained Monolayer MoS_2 and WS_2. *Annalen der Physik,* 2014, *526* (9–10), L7–L12.

[s24] Carrascoso, F., Lin, D. Y., Frisenda, R., & Castellanos-Gomez, A. Biaxial strain tuning of interlayer excitons in bilayer MoS2. *Journal of Physics: Materials*, 2019, 3(1), 015003.

[s25] Biron, M. (2018). Thermoplastics and thermoplastic composites. Thermoplastic Composites, *William Andrew Publishing*, 2013, Pages 769-829

[s26] Gupta, S., Dixit, M., Sharma, K., & Saxena, N. S. Mechanical study of metallized polyethylene terephthalate (PET) films. *Surface and Coatings Technology,* 2009, 204(5), 661-666.

[s27] Li, Z., Chen, X., Zhang, C., Baer, E., Langhe, D., Ponting, M., ... & Zhu, L. High dielectric constant polycarbonate/nylon multilayer films capacitors with self-healing capability. *ACS Applied Polymer Materials*, 2019, 1(4), 867-875.
